# Investigation on the Influence of Protein Corona and Platelet Adhesion on Storage Bag Surface on the Platelet Storage Lesion

**DOI:** 10.1002/adhm.202501217

**Published:** 2025-06-23

**Authors:** Nicolas Pereyra, Kai Yu, Jason Rogalski, Taylor Olivia Da Silva, Parisa Golesorkhi, Madeleine Goldstein, Dana V. Devine, Jayachandran N. Kizhakkedathu

**Affiliations:** ^1^ Centre for Blood Research and Life Sciences Institute University of British Columbia 2350 Health Sciences Mall Vancouver BC V6T 1Z3 Canada; ^2^ Department of Biochemistry & Molecular Biology University of British Columbia 2350 Health Sciences Mall Vancouver BC V6T 1Z3 Canada; ^3^ Department of Pathology and Laboratory Medicine University of British Columbia 2350 Health Sciences Mall Vancouver BC V6T 1Z3 Canada; ^4^ Centre for High Throughput Biology Michael Smith Laboratories University of British Columbia University of British Columbia 2125 East Mall Vancouver BC V6T 2A1 Canada; ^5^ Department of Cellular and Physiological Sciences University of British Columbia University of British Columbia 2350 Health Sciences Mall Vancouver BC V6T 1Z3 Canada; ^6^ School of Biomedical Engineering University of British Columbia Vancouver BC V6T 1Z3 Canada

**Keywords:** antifouling coatings, platelet storage, platelets, polydopamine, polymer coatings, protein corona

## Abstract

Platelet transfusion is an indispensable therapy used in contexts ranging from hemorrhagic bleeding to chemotherapy. However, platelet shelf life and quality deteriorate during storage in a process termed the platelet storage lesion (PSL), resulting in chronic shortages globally. The PSL is thought to be partially attributed to the bio‐incompatible polyvinyl chloride (PVC) storage bags that promote protein fouling and platelet adhesion. Developing platelet‐friendly materials is therefore essential for improving platelet storage quality. This study aimed to delineate the contributions of protein adsorption and platelet adhesion to the progression of the PSL. Hydrophilic coatings are screened to identify those which resist protein and platelet adhesion most effectively. These coatings are translated into mini‐platelet bags and tested in long‐term standard blood banking conditions. Significant reductions in platelet adhesion after 7‐day storage do not affect platelet quality. Proteomic characterization of the bag surfaces revealed dynamic changes in the protein coronas on coated and uncoated bag over time, but are uncorrelated with platelet quality. This research demonstrates a method to identify platelet‐compatible coatings suitable for long‐term storage, as well as a novel approach to characterize the surface of blood storage bags.

## Graphical abstract

Platelet quality deteriorates during storage due to protein and platelet adhesion to current bag materials. Here, polymer coatings are identified that reduce adhesion and translated them to mini‐storage bags. Platelet quality are not impacted, despite significantly reducing adhesion. Surface protein profiles varied across materials but showed no correlation with platelet quality, suggesting other factors drive the deterioration of platelet quality.

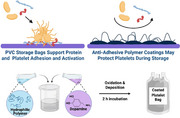

## 1 Introduction

Platelets are blood cells involved in hemostasis, coagulation, healing, and inflammation.^[^ ^1^ ^]^ Platelet concentrates (PC) are indispensable transfusion products in medical care and widely used in life‐saving therapies ranging from controlling severe bleeding to disorders involving genetic or acquired thrombocytopenia, such as chemotherapy.^[^ ^2^ ^]^ Currently, PCs are stored in storage bags made from polyvinyl chloride (PVC) with plasticizers and are kept under constant gentle agitation at 22 ± 2 °C for 5 days, and up to 7 days in Canada.^[^ ^3^ ^]^ However, throughout processing and storage, platelets undergo a set of biochemical and functional changes including activation, aberrant morphology, reduced aggregation and clotting capacity, apoptosis, and dysregulation of the mitochondria.^[^ ^4^, ^5^ ^]^ Collectively, these changes are referred to as the platelet storage lesion (PSL). Throughout storage, this deterioration reduces the quality of the cells, leading to inferior transfusion outcomes.^[^ ^6^, ^7^ ^]^ The PSL also shortens platelet lifespan from 8–10 days in circulation to a maximum shelf life of 5–7 days.^[^ ^4^, ^5^ ^]^ Due to the curtailed shelf‐life and transfusion quality of the platelets, the PSL remains a critical and challenging management problem for blood services globally.^[^ ^8^, ^9^, ^10^, ^11^ ^]^ Thus, improving the lifespan and storage quality of platelets is an essential step to increase blood product supply and clinical outcomes.

Numerous factors contribute to the emergence of the PSL, including manipulation during manufacture, storage under continuous agitation, oxidative stress, time in storage, and the accumulation of metabolic by‐products.^[^ ^4^, ^12^, ^13^, ^14^, ^15^ ^]^ The storage bag, their composite materials, and bag material surface interactions with platelets and proteins are also thought to play an important role in the PSL.^[^ ^16^, ^17^, ^18^ ^]^ Plasticized PVC has been widely adopted as the material of choice in many blood‐contacting devices, including platelet storage bags, for its durability, flexibility, and relative compatibility with blood components. Despite its strengths, plasticized PVC has deleterious effects on stored platelets: its hydrophobic surface promotes protein fouling due to elevated surface free energy when in contact with aqueous solutions.^[^ ^19^ ^]^ In plasma, PVC supports the adhesion, conformational change and activation of plasma proteins, including complement and coagulation proteins (e.g., fibrinogen).^[^ ^15^, ^20^, ^21^, ^22^, ^23^, ^24^ ^]^ This protein fouling enables both platelet and bacterial adhesion, as well as biofilm formation.^[^ ^22^, ^23^, ^24^ ^]^ Platelets which come in contact with these deposited proteins can adhere and become activated, releasing their granular contents and exposing surface integrins that activate surrounding platelets.^[^ ^20^, ^25^, ^26^ ^]^ The plasticizers employed with PVC can also affect platelet storage by leaching and altering the bag surface's hydrophobicity and gas permeability, thereby contributing to the PSL.^[^ ^17^, ^27^, ^28^, ^29^, ^30^ ^]^ As such, developing biocompatible materials for platelet storage bags that can combat the negative effects of plasticized PVC may improve storage quality and shelf life.

Despite the importance of PCs in modern medicine, surprisingly little research has been published on improving materials for platelet storage due to the challenges of working with human platelets and applying novel materials onto bags. Published research has scarcely tested these novel surfaces in platelet storage bags, opting instead to employ test tubes, platelet bag cut‐outs (coupons) in well plates, or other model storage vessels to evaluate the platelet compatibility^[^ ^14^, ^16^, ^18^, ^31^, ^32^, ^33^ ^]^; these approaches include nanopatterning of the surfaces and hydrophilic antifouling coatings, etc. (approaches previously reviewed^[^ ^26^, ^34^, ^35^, ^36^ ^]^). Working with platelets over longer timepoints is also a well‐known challenge due to their intolerance of many common lab tools and techniques, including plastic tubes and well plates.^[^ ^37^, ^38^, ^39^ ^]^ Even different volumes of PCs used can impact cell quality due to changes in fluid mechanics and gas permeability.^[^ ^39^, ^40^ ^]^ For those studies which employ platelet storage bags, plasticizers are an important consideration as well, as plasticizers like di(2‐ethylhexyl) phthalate (DEHP) can significantly impact cell quality due to a combination of hydrophobicity, leaching plasticizers, and gas permeability.^[^ ^17^, ^29^, ^32^, ^41^ ^]^ These confounding effects make translating prototypes from the benchtop into platelet bags difficult. In addition, little data is available on microstructural changes, adsorption sites, and binding kinetics of plasma proteins on surfaces: Such studies can be performed on isolated proteins, and molecular simulations can model a small number of interactions as well. However, no approaches to date can simulate or determine the interactions between, and changes to, proteins in the highly complex plasma protein corona, nor the myriads of resulting pathways which might be initiated in platelets and plasma as a result. Indeed, despite the importance attributed to protein adsorption on platelet bags, an investigation into the proteome formed on platelet bag surfaces is not yet available. Because of these challenges, it is unclear which approaches are most effective in improving storage bag biocompatibility, combatting the PSL, which variables in simplified systems are the most representative of true improvements to platelet storage quality, and how consistent these effects are upon scale‐up and translation.

Polydopamine (PDA)‐based antifouling polymer coatings are a common approach to develop surfaces capable of resisting cell adhesion and protein adsorption.^[^ ^42^ ^]^ These antifouling coatings resist protein adsorption and cell adhesion by establishing a hydration layer over the substrate surface, imposing a free‐energy penalty that prevents adsorption to the underlying PDA and substrate. The coatings can be tuned to resist protein and cell adhesion further by using hydrophilic polymers with different chemistries (zwitterionic, amphipathic, etc.), as well as by altering their abundance relative to PDA, and changing their molecular weights.^[^ ^43^, ^44^, ^45^ ^]^ PDA‐hydrophilic polymer coatings can also be adapted to coat many materials, regardless of surface chemistry or topology, offering a practical approach to modifying the surface of platelet storage bags.^[^ ^44^, ^46^, ^47^ ^]^

The literature data suggest that a key factor leading to deterioration in platelet quality is the unfavorable interactions between platelets and the non‐biocompatible hydrophobic storage bags, which cause irreversible plasma protein binding and denaturation, platelet adhesion, and activation.^[^ ^15^, ^22^ ^]^ However, there is very limited data available on the contribution of these factors to long‐term platelet storage quality and PSL. Thus, in this research, we have developed platelet friendly hydrophilic PDA‐polymer coatings on platelet storage bags which minimize the protein adsorption and platelet adhesion. We leveraged the tunability of these coatings to screen a library of polymer candidates to identify the optimal anti‐adhesive platelet friendly coating compositions. Top‐performing coating chemistries were then translated into platelet bags and tested under blood banking conditions for long‐term storage quality of human platelets in this storage bags (until day 7) including the measurements of activation, platelet morphology, coagulation profile, blood gas and platelet adhesion. Further, using proteomics analyses, for the first time, we further dissected the roles of adsorbed protein corona formed on the surface of the platelet bags with and without coating during storage and their link with PSL.

## 2 Results

### 2.1 Screening of Antifouling Polymer Coating Compositions on Platelet Bag Coupons against Platelet Adhesion

**Figure 1:**
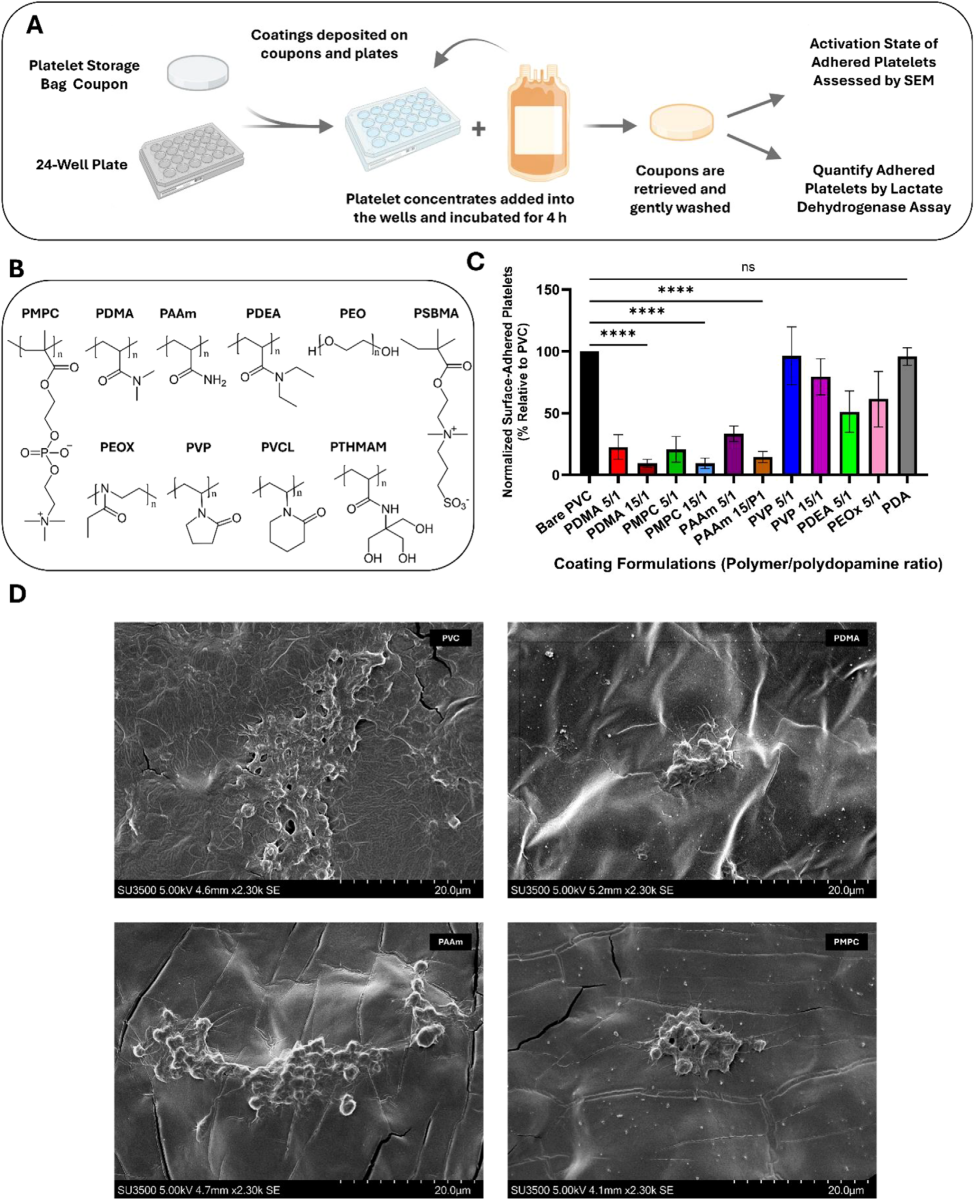
Screening of anti‐adhesive coatings for platelet storage bags. A) Schematic of the experimental workflow to generate a library of PDA/hydrophilic polymers coatings and test their anti‐adhesive properties in PCs. B) Chemical structures of the library of hydrophilic polymers screened to develop anti‐adhesive platelet storage bags. C) Quantification of platelet adhesion onto different coating compositions after 4 h‐incubation by lactate dehydrogenase (LDH) assay. Values are shown as % binding relative to the number of platelets adhered on PVC coupons. D) Representative images of the adhered platelets were taken by SEM for PVC, and different coatings containing PDMA, PAAm, and PMPC. The ratio of the polymer to PDA ratio is shown in X‐axis labels. Statistical significance is denoted by ^*^ system: (^*^) p<0.05, (^**^) p<0.01, (^***^) p<0.001, ^****^ p<0.0001. N = 3 independent biological replicates for all figures shown. Panel A prepared using Biorender.com.

Our initial effort was to identify the optimal platelet friendly polymer coating compositions that could resist platelet adhesion from human platelet‐rich plasma for application in platelet bags. One of our other goals was to develop a coating composition that can be easily applied to platelet bags in aqueous solution without affecting the properties of plasticized PVC. Thus, coupons were firstly cut from industry‐standard PVC platelet storage bags (Tris(2‐ethylhexyl) trimellitate, TOTM)‐plasticized PVC) to evaluate the coating formation and the efficiency of the coating in reducing platelet adhesion on the coating in comparison to uncoated bag coupons as a benchmark (**Figure** **1**A). The ability of the coatings to resist adsorption of fibrinogen and human serum albumin (HSA) was also tested. We utilized substrate‐independent hydrophilic polymer‐assisted PDA assembly and deposition technique in aqueous buffer to develop diverse polymer coatings on plasticized PVC surface.^[^ ^44^ ^]^ The coating technique allowed us to use PDA as a surface binder for the hydrophilic polymers that assemble to form a stable and highly hydrophilic coating layer on the surface of PVC coupons.^[^ ^44^, ^45^ ^]^ A diverse library of ultrahigh molecular weight hydrophilic polymers (UHHP) with different hydrophilicity and molecular weights in combination with dopamine were used for the generation of hydrophilic coating on PVC surface for this initial screening (Figure 1B). The dopamine concentration was kept as 2 mg mL^−1^, while the concentrations of the hydrophilic polymers were altered from 10 to 30 mg mL^−1^, yielding final ratios of polymer:PDA from 5:1 to 15:1.

To evaluate the platelet adhesion‐resistance of various coating compositions, the coated PVC coupons were incubated with human platelet rich plasma (PRP) for 4 h at 22 ± 2 °C kept under gentle agitation to avoid platelets settling out of suspension. The efficiency of the coating in reducing platelet adhesion was measured by both LDH assay and SEM.^[^ ^16^ ^]^ Representative data on the adsorption of HSA and fibrinogen is shown in Figure S1 (Supporting Information), which show significant reduction in the adsorption of these proteins on coated surface. Figure 1C shows the platelet adhesion onto different hydrophilic polymer/PDA coating. Among all the polymers investigated, poly (N,N‐dimethylacrylamide) (PDMA), poly (2‐methacryloyloxyethyl phosphorylcholine) (PMPC) and polyacrylamide (PAAm) showed better efficiency up to 90% in reducing platelet adhesion. The higher content of polymers in the coating (15:1 to 5:1) is helpful for further enhancing its anti‐adhesion properties (Figure 1C). Adhesion of the platelets was further examined by scanning electron microscopy (SEM) (Figure 1D).^[^ ^16^ ^]^ Platelet aggregates were easily identified on the bare PVC coupons, consistent with increased adhesion of platelets measured by the LDH assay, whereas platelets on all coated surfaces were scarcely visible. Aggregates composed of adhered platelets and other structures (e.g., fibrin or cell debris) were visible on all surfaces. However, those on PVC were the largest, containing >50 platelets in the structures, with many other solitary platelets scattered in the vicinity. Morphologically, platelets on PVC showed extensive spreading, filopodia extension, and few maintained their sphericity. Aggregates were smaller on all coated surfaces, with PAAm containing the most (20 to 30 platelets per aggregate), while PDMA and PMPC had the smallest (≈10 platelets per aggregate), and no solitary platelets were seen. Platelets adhered to the coatings also showed spreading and filopodia extension, but most maintained a partly spherical shape. Representative data on the adsorption of albumin and fibrinogen is shown in Figure S1 (Supporting Information), where a significant reduction in the adsorption of these proteins on coated surface was seen. The PDMA, PAAm, and PMPC coatings in 15:1 ratio with PDA reduced fibrinogen binding > 99.5% for all coatings, and HSA by >99%. Based on these data, we have selected coating compositions of 15:1 (hydrophilic polymer:PDA) for PDMA, PMPC and PAAm polymersfurther studies on developing antifouling mini‐platelet storage bags.

### 2.2 Development of Protein and Platelet Repelling Mini‐Platelet Bags and Platelet Storage Quality Analysis

**Figure 2:**
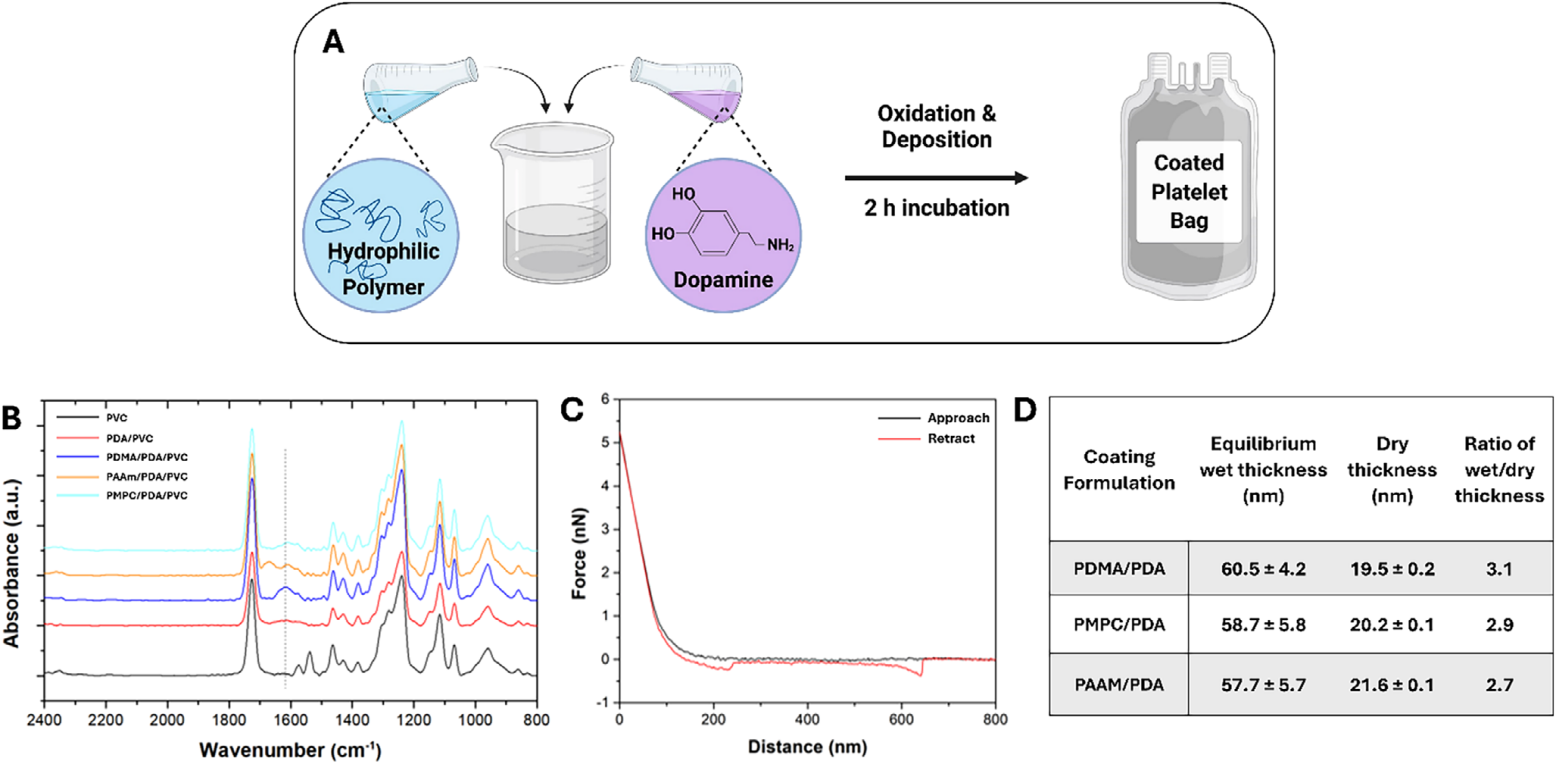
Workflow and characterization of UHHP‐PDA coating deposited on mini‐platelet storage bags. A) Mini‐PVC platelet bags (45 mL, Figure S2A, Supporting Information) were coated with one of 15:1 PDMA, PAAM, or PMPC:PDA. To coat the bags, the polymer and PDA solution were introduced into the bags and the reaction was initiated by adding 0.2 mg mL^−1^ NaIO_4_. B) Attenuated total reflection fourier transform infrared (ATR‐FTIR) spectra of hydrophilic polymer/PDA coating on the PVC bag surface. C) Atomic force microscopy (AFM) measurements  of the coatings prepared in the bags by the co‐assembly of PDA with PDMA, on the PVC surface. The black line indicates the approach curve, and the red line indicates the retraction curve. D) Table showing the wet (aqueous buffer) and dry thicknesses of the coatings deposited in the mini‐ platelet storage bags used in the study. N = 3 independent experiments. Panel A prepared using Biorender.com.

**Figure 3:**
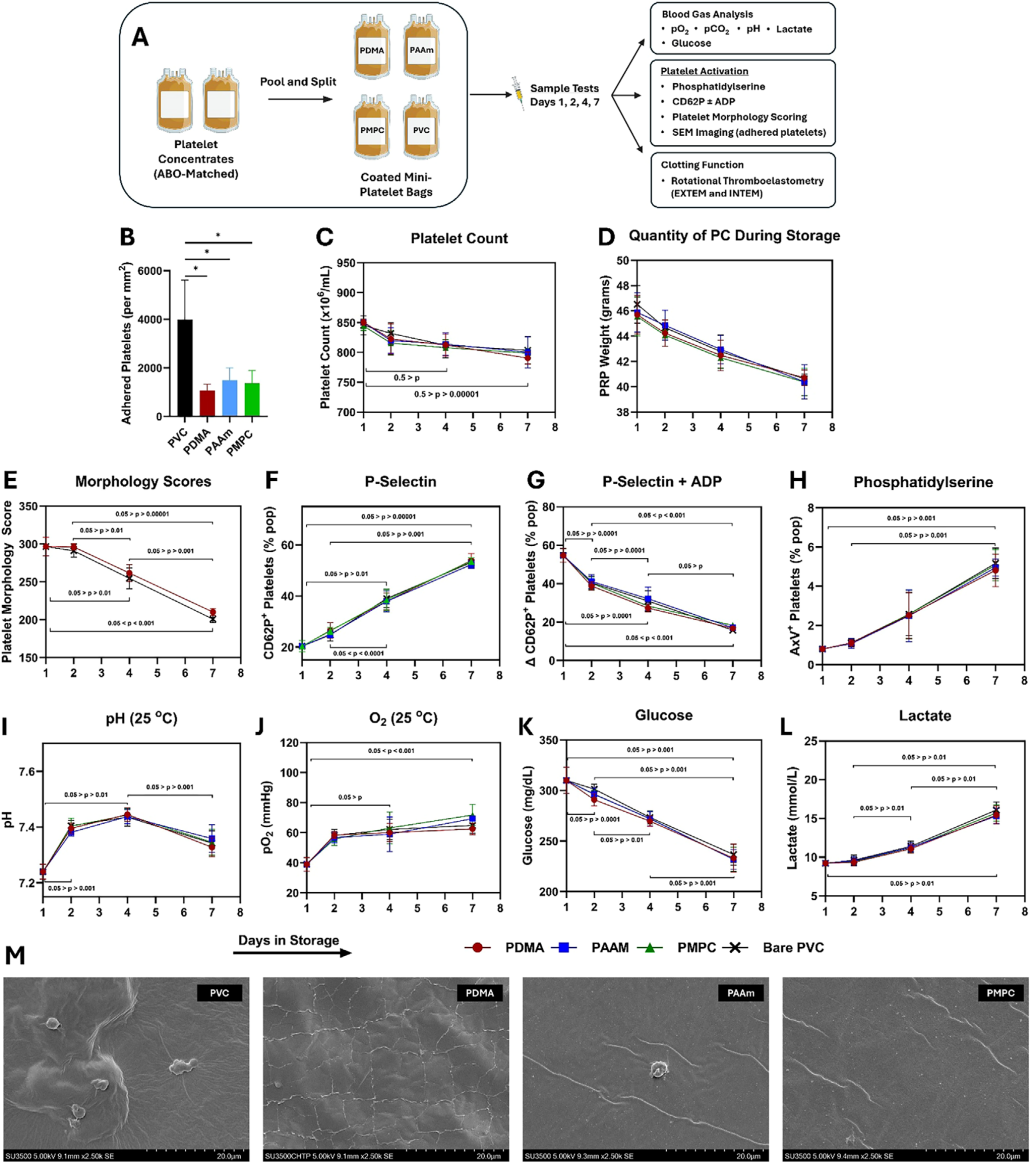
Comparison of platelet storage quality in PVC and polymer‐PDA coated bags. A) Mini‐PVC platelet bags (45 mL, Figure S2A, Supporting Information) were coated with one of 15:1 PDMA, PAAM, or PMPC:PDA, or left uncoated (bare PVC). Two pooled buffy coat PCs in plasma were pooled‐and‐split as described (see methods for details).^[^ ^52^, ^53^ ^]^ The pooled PCs were then separated into coated bags (technical triplicates for each) and placed into storage. On days 1, 2, 4, and 7, PCs were evaluated using a set of industry‐standard methods. B) Number of platelets adhered to the bag surfaces after 7‐day storage. C) Platelet counts in the storage bags throughout 7‐day storage. D) Volume of PC in the storage bags throughout the study. Data shown as mean ± SD. E) Kunicki platelet morphology scoring.^[^ ^54^ ^]^ F) Surface‐display of CD62P. G) Platelet responsiveness was evaluated by measuring the rise in CD62P after activation of the platelets with 10 µm ADP. H) Surface‐displayed phosphatidylserine by annexin V staining. I–L) Metabolic health throughout storage was tracked by measuring pH, O_2_, glucose, and lactate, respectively. M) Electron micrographs of the platelets adhered on the various storage bag surfaces after 7‐day storage. Scale bar shows 20 µm. PDMA (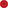), PAAM (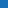), PMPC (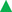), Bare PVC (X). No significant differences were found between bag compositions. Significant differences within coatings across time are marked by the pairwise comparisons. The figure above shows the range of the p values in the corresponding comparisons to clarify the figure. For a full list of the detailed comparisons, see Table S2 (Supporting Information). N = 3 independent biological replicates for each. Panel A prepared using Biorender.com.

The data collected from the screening (Figure 1; Figure S1, Supporting Information) using coated PVC coupons indicated that the hydrophilic polymer coating could diminish platelet adhesion and their subsequent activation, as well as reduce protein adsorption. The lead polymer coatings were subsequently translated into platelet storage bags for the investigation of platelet storage. The surface to volume ratio of the mini‐platelet storage bags (45 mL, Figure S2A, Supporting Information) are similar to that of standard PC storage bags used in blood banks. We used TOTM‐PVC for the development of the bag for its reduced cytotoxicity and leaching when compared to DEHP.^[^ ^48^, ^49^ ^]^ The insides of mini‐platelet storage bags were coated with the lead polymer compositions (PDMA, PAAm, and PMPC in 15:1 ratio with PD) identified in Figure 1, as detailed in the Methods section (workflow and results shown in **Figure** **2**). The coated and uncoated mini‐platelet storage bags were then sterilized using 70% ethanol followed by washing with sterile 10 mm phosphate buffered saline (PBS) before introduction of pooled human PCs (≈900 × 10^6^ platelets mL^−1^) suspended in plasma. PCs were prepared by CBS using standard protocols for pooled buffy coat platelets generated from 4 donors per PC (**Figure** **3**A). For these studies, PCs were prepared by pooling‐and‐splitting 2 ABO‐matched pooled buffy coat platelets (4 donors’ platelets in each unit, for a total of 8 donors’ platelets per biological replicate), using our previously published methods, enhancing the control of donor variability in this study.^[^ ^50^, ^51^, ^52^ ^]^

For the long‐term platelet storage study, mini‐platelet storage bags (Figure S2A, Supporting Information) were coated with the 3 formulations PDMA, PAAm, and PMPC 15:1 with PDA, as shown in Figure 2A. The coated bags were characterized for uniformity of the coating and hydrophilicity: Figure 2B shows representative ATR‐FTIR spectra of the polymer coating prepared from PDMA, PAAm, and PMPC with PDA (15:1 ratio). The incorporation of polymers within the coating was evidenced by the prevailing peaks at 1621, 1614, 1612 cm^−1^, owing to the adsorption of carbonyl group stretching (amide I band) in the PDMA, PAAm and PMPC (Figure 2B). The application of coating on the PVC rendered the surface hydrophilic (Figure S3, Supporting Information) for example, the water contact angle decreasing from 93° for the unmodified PVC to 32°, 14°, 18° for the PDMA/PDA, PAAm/PDA and PMPC/PDA coatings respectively. The coating was further characterized using AFM analysis.^[^ ^16^ ^]^ Representative force curves of coated samples versus bare PVC are shown in (Figure 2C) suggesting that the hydrophobic PVC is fully covered by the polymer coating.^[^ ^16^ ^]^ From the force profile, the wet thicknesses of the PDMA/PDA, PMPC/PDA and PAAm/PDA coatings were calculated and are given in Figure 2D. The wet thickness of the coatings was ≈2.7‐3.1‐fold higher than the dry thickness indicates that the polymer chains in the coating is hydrated and stretched away from the underlying surface. The approach force curve (Figure 2C) also supports this.^[^ ^44^ ^]^ With this confirmation that the coatings were uniformly deposited in the platelet mini‐storage bags, we then evaluated the quality of platelet storage with these modified mini‐storage bags.

The experimental design for platelet storage in mini‐platelet bags in shown in Figure 3A. The bags were filled with pooled‐and‐split human PCs (8 donors per master PC, see methods for details) and placed into blood bank‐standard platelet shakers and stored at 22 °C for 7 days. The pooled PCs were received on day 1 (before the start of experiment), placed into different mini‐bags, and quality was assessed on day 2, day 4 and day 7. Figure 3C,D gives the starting parameters for each of the platelet conditions, as well as the changes to the platelet counts and changes to bag volumes throughout the storage study.

The platelet storage quality was assessed by measuring several parameters used in standard blood‐banking practice (Figure 3) at different timepoints. Figure 3E‐L shows these quality metrics throughout storage, and Figure 3M shows SEM photos of the platelets adhered to the mini‐bags after 7‐day storage. After 7 days of storage, all three coating formulations significantly reduced the number of adhered platelets on the surfaces (PDMA > 60%, PMPC and PAAm > 50%, Figure 3B), consistent with the data generated using PVC coupons. SEM images of the bags after 7 days (Figure 3M) showed adherent platelets had similar morphology on all bags tested, marked by loss of discoid shape and extension of filopodia. Platelets on the PVC bags were easily identifiable, and many aggregates and debris were present as well. Conversely, platelets were difficult to locate on the coated surfaces, and few aggregates and debris were visible. While the adhered platelets on all the surfaces showed shape‐change, no differences were apparent between the cells in different bag coating formulations. The platelet quality metrics indicate deterioration of the cells throughout the storage period (Figure 3E–L), in line with the PSL. For platelet activation states, significant increases in P‐selectin and phosphatidylserine (Figure 3F,H) were seen between days 1–2 and day 7, while platelet morphology and responsiveness (Figure 3E,G) decreased. Metabolic markers of platelet quality (pH, O_2_, glucose, and lactate, Figure 3I–J respectively) began to worsen slowly, before meeting an inflexion point at day 4, after which deterioration accelerates, which agrees with previous metabolomic analyses of the PSL's timeline.^[^ ^12^ ^]^ Platelet count decreased steadily across time (Figure 3C), with a significant decrease between days 1 and 7 for PDMA and PMPC, as well as between days 2 and 4 for PVC. It should be noted that platelet volume remained within 15% of the initial volume (Figure 3D) throughout the experiment. Interestingly, despite these changes across time, no differences were found between the platelets stored in the various bags irrespective of the nature of the polymer coatings or coated or uncoated bags.

**Figure 4:**
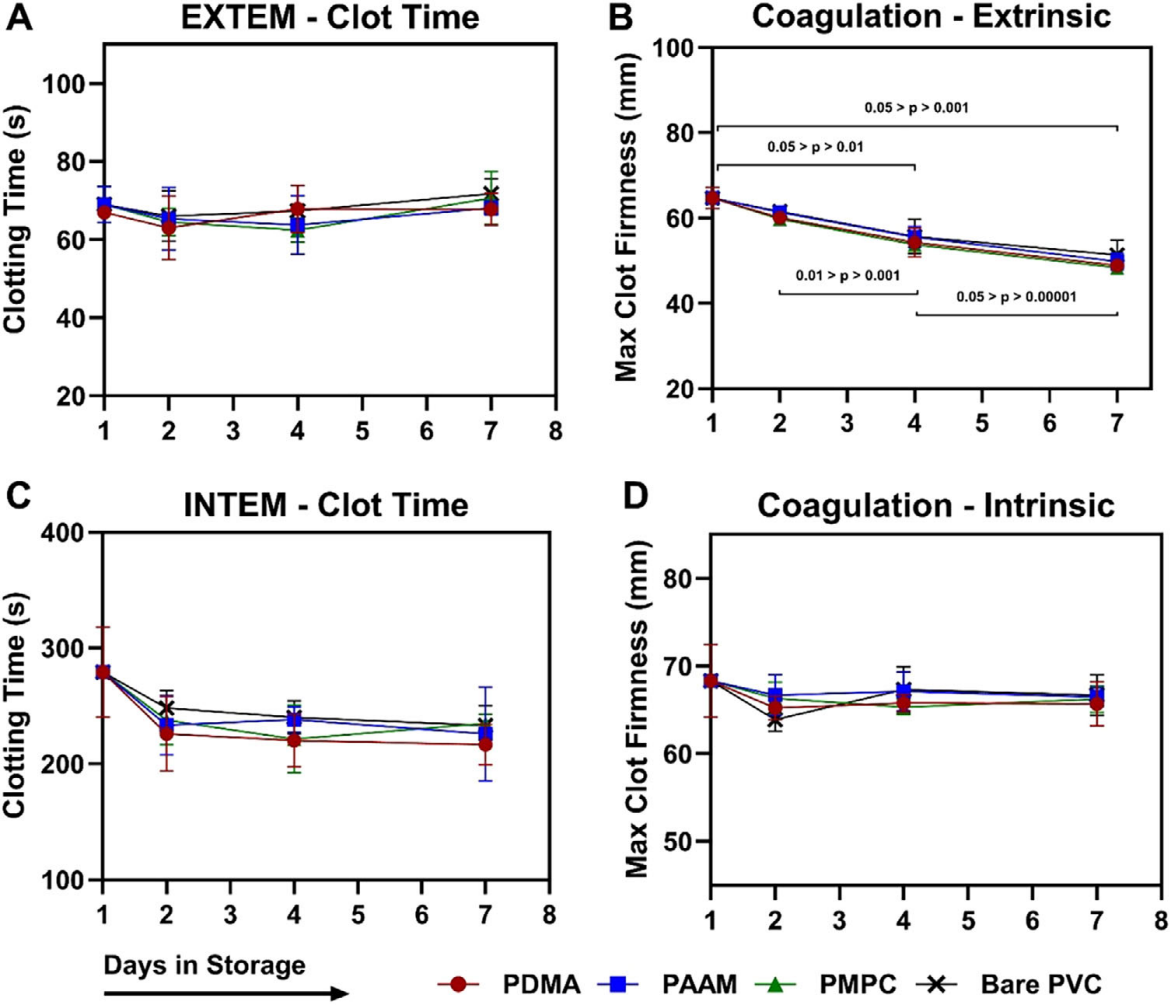
ROTEM analysis of platelets in anti‐adhesive storage units. ROTEM reactions were initiated with START‐TEM reagent, as well as either EX‐TEM A,B) or IN‐TEM (C and D). (A) EX‐TEM Clotting Time. (B) EX‐TEM MCF, Maximum Clot Firmness. C) IN‐TEM Clotting Time. D) IN‐TEM, MCF. Results are expressed as mean ± SD. Legend: PDMA (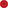), PAAM (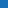), PMPC (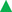), Bare PVC (X). No statistical differences were found between any of the coatings within a given timepoint. Significant differences within coatings across time are marked by the pairwise comparisons. The range of the p‐values found are noted above the corresponding comparisons. For a full list of significant differences, see Table S2 (Supporting Information).

Coagulation dynamics of the stored platelets were investigated using rotational thromboelastometry (ROTEM) (**Figure** **4**).^[^ ^55^ ^]^ Max clot firmness when initiated by EXTEM significantly decreased throughout storage for all coating conditions (Figure 4B). Conversely, platelet clotting time when activated by EXTEM and INTEM, as well as INTEM max clot firmness, did not significantly differ across time. As seen in Figure 4, no significant differences were found between the platelets stored in the different coated bags here either.

### 2.3 Proteomic Characterization of Protein Corona in Coated and Uncoated Platelet Storage Bags

The adsorbed protein corona from blood plasma represents the new surface that cells and biological molecules encounter at the interface of materials and biological fluids.^[^ ^56^ ^]^ The hypothesis was that the changes in adsorbed protein corona could contribute to the changes in the platelet storage behavior and platelet storage lesion. Thus, to further understand the performance of the coated and uncoated mini‐platelet bags, we investigated the protein corona on bags at different timepoints. To investigate the protein corona formed on the surface, PVC‐TOTM 45 mL mini‐bags were either coated with PDMA 15:1 PDA or left uncoated as controls. Human platelet poor plasma was filled, and the protein corona changes were measured over the storage period. The adsorbed proteins were in situ digested and analyzed by mass spectrometry. Proteins were identified by library‐free search on DIA‐NN using a Homo sapiens database from Uniprot and were quantified by high precision cross‐run normalization for library generation.

**Figure 5:**
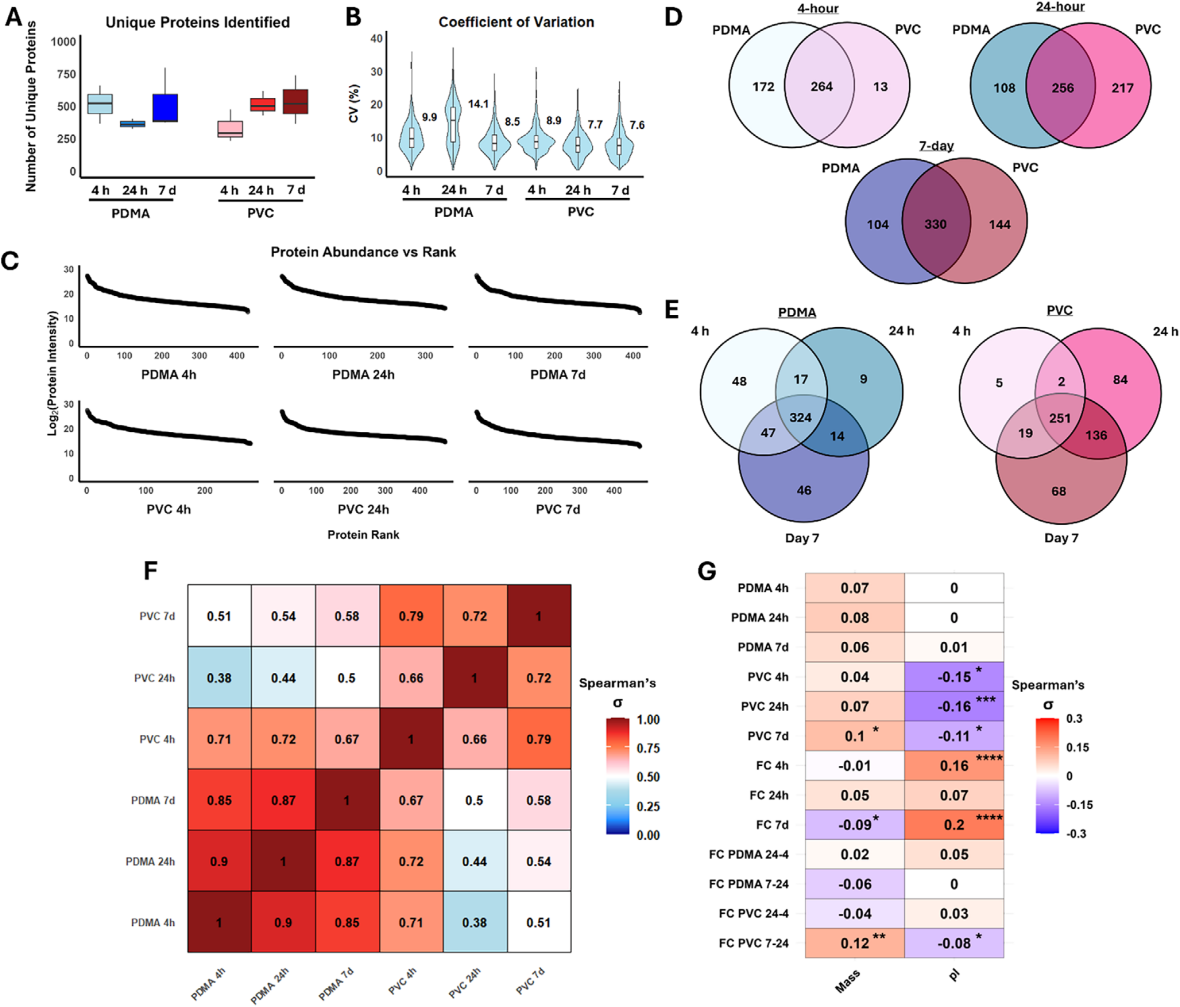
Overview of the proteomics data on uncoated PVC and PDMA‐coated platelet storage bags. A) Number of unique proteins identified per sample on the surface of PDMA‐coated and uncoated PVC platelet storage bags before applying inclusion‐exclusion criteria (detected proteins in at least 2 of 3 biological replicates within a given timepoint and condition). B) Coefficients of variation of log_2_‐transformed protein intensities after inclusion‐exclusion criteria. Figure shows the average of the CVs calculated for each protein within the timepoint and condition. C) Scatterplots showing the abundance of each protein, and their corresponding rank within each dataset. One point for each protein. Most abundant proteins are shown on the top left, least abundant on the bottom right. D) Comparison of unique and common proteins between PDMA‐coated and PVC‐coated bags at 4, 24 h, and 7d. E) Comparison of unique and common proteins on PDMA and PVC across timepoints. F) Correlation matrix of protein abundance across conditions and timepoints, calculated using Spearman's correlation test. Cell colors are based on the size of the σ, with exact values for each comparison pasted within. All p values were <0.0001. G) Correlation matrix of protein abundance with respect to the mass and isoelectric point (pI) of the peptides, calculated using Spearman's correlation. The groups being compared are listed on the left side of the figure. Cells are colored based on the size of the σ, with exact values pasted within. Row legend: Correlation of individual coatings and timepoints is labelled. Fold changes of PDMA coating or uncoated PVC were noted as “FC” with the corresponding timepoint. Fold changes across timepoints within a condition were denoted as “FC material” with the corresponding timepoints. Statistical significance is denoted by ^*^ system: (^*^) p<0.05, (^**^) p<0.01, (^***^) p<0.001, ^****^ p<0.0001. N = 3 independent biological replicates for all figures shown.

The proteomes were first analyzed for holistic changes across time and conditions (**Figure** **5**). Proteins met the inclusion criteria if detected in all technical replicates and at least 2 of 3 biological replicates; Figure 5A shows boxplots for the resultant protein counts. This approach yielded ≈450 unique proteins for each condition (Figure 5A). Protein counts for PVC were lowest at 4 h and increased with time, while PDMA remained consistent throughout. Both surfaces hosted the highest number of unique proteins at 7 days (≈500 each). Given the stochastic nature of plasma proteomics the relatively low abundance of proteins that met the inclusion criteria,^[^ ^57^ ^]^ the coefficient of variation (CV) of the log2 intensities for each protein was calculated across all biological replicates within a given timepoint and condition, and the average CV for each dataset was determined (Figure 5B). The average CV of the protein intensities was ≈8.5, except for PDMA coated bag at 24 h with 14.5 (Figure 5B). To compare the skewedness of the total protein intensity toward the most abundant proteins, the detected intensities were plotted against the average rank of the proteins within each condition (Figure 5C). Here, protein distribution was skewed toward the top 100 highest‐intensity proteins. This skewedness increased with storage time, particularly for the top 50 proteins on uncoated PVC bags at 24 h and 7 days. For PDMA bags, the protein intensity distribution rose more gradually across ranks and time (Figure 5C).

The dynamics of unique protein numbers, abundances, and overlap were subsequently investigated, revealing clear differences between PDMA‐coated and bare PVC bags (Figure 5D‐G). Figure 5D demonstrates the commonality of the proteins detected between the bags at each timepoint: at 4 h, 60% and 95% of the proteins were shared between PDMA coated bag and uncoated PVC at 4 h (respectively), attributed to the low number of unique proteins on uncoated PVC at 4 h. However, over time, PDMA coating's commonality with uncoated PVC increased (70% and 76% at 24 h and 7d, respectively), whereas the proportion of unique proteins on PVC increased as well (55% for 24 h and 70% for 7d, respectively, Figure 5D). Figure 5E shows the commonality of the proteins on each surface across time: here, uncoated PVC had the most unique proteome at 24 h (18% unique, compared to 2% and 14% at 4 h and 7d, respectively). Conversely, PDMA coating was the least unique at 24 h (2%).

The matrix of Spearman's correlation tests comparing the abundance of proteins detected in each condition further support these trends (Figure 5F): uncoated PVC showed the lowest inter‐timepoint correlation at 24 h (σ = 0.66 and 0.72 with 4 h and 7 d, respectively), whereas PDMA coating's protein abundances were consistent across time (average σ = 0.87) compared to PVC (average σ = 0.72) (Figure 5F). Despite these differences, the trends were not associated with the mass or pIs of the proteins detected (Figure 5G): A weak correlation was identified between the abundance of the proteins and mass for all conditions, especially for uncoated PVC bags over time (Figure 5G). However, correlation of the fold changes across time and between conditions did not reveal any associations between mass and the accumulation patterns seen. While significant but weak correlations (Spearman's σ < 0.3, Figure 5G) were found between pIs and the fold change of PDMA coating and uncoated PVC, due to the pI/pH hypothesis, this may be attributed to the fact that most plasma proteins have a pI below 7.^[^ ^58^, ^59^ ^]^

To contextualize the differences in the percentage compositions of the proteomes, the amounts of proteins desorbed from each surface and timepoints were measured (Figures S4 and S5, Supporting Information). Approximately 4 mg of proteins were eluted from each bag surface across the timepoints for both PDMA coated bag and uncoated PVC bag (Figure S5, Supporting Information), and no significant differences were found in the amounts of proteins eluted.

**Figure 6:**
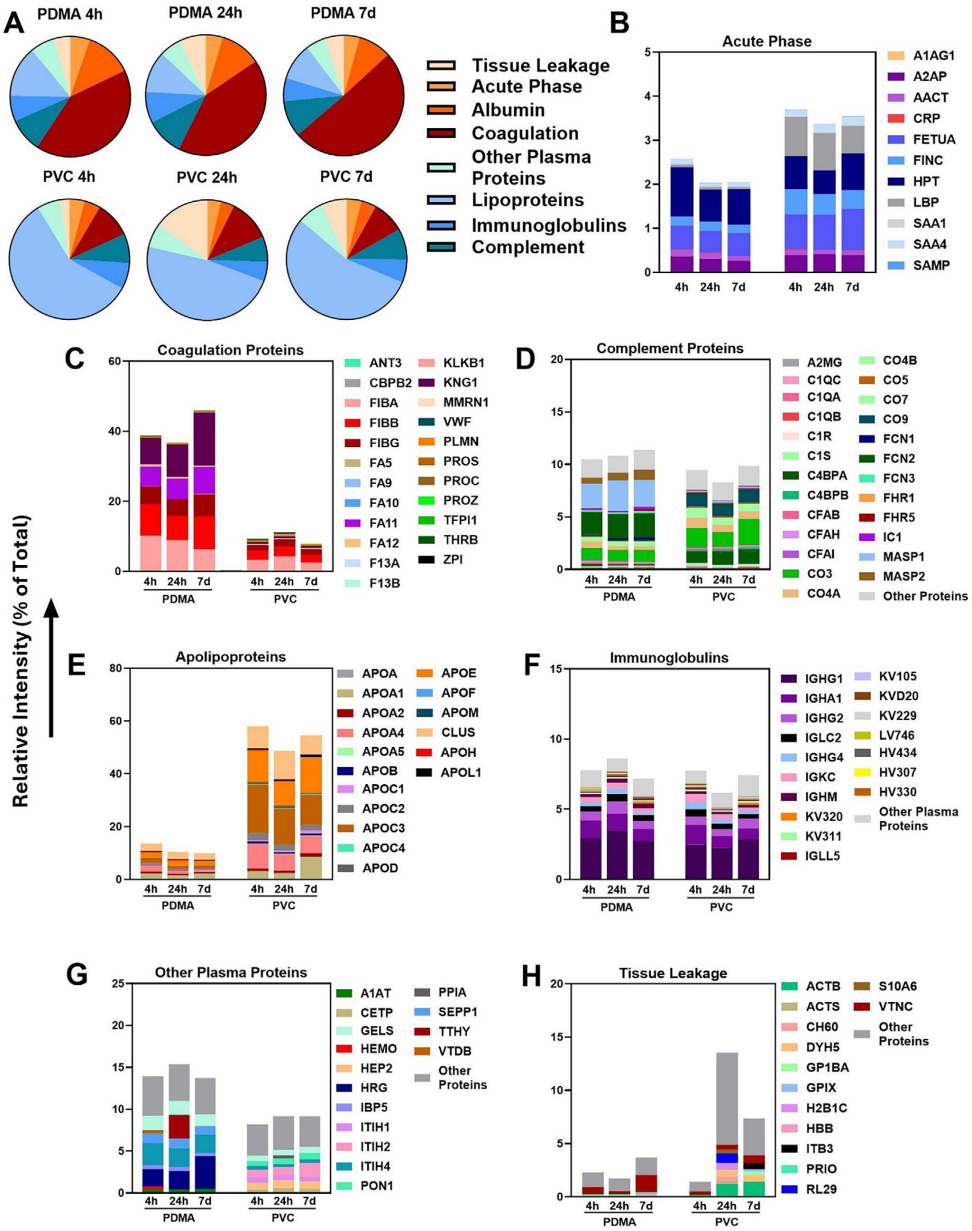
Analysis of protein corona formed on the PDMA/PDA‐coated and uncoated mini‐PVC platelet storage bags over 7 days. Proteins identified on PDMA/PDA coated bag and uncoated PVC bags at 4, 24 h, and 7 days were classified based on biological function. A) Abundance of proteins by biological function group as a fraction of the total proteins identified in the corona at each timepoint and condition. The abundance of the individual proteins in the corona, classified based on their biological function, are shown as B) acute‐phase reactants, C) coagulation proteins, D) complement proteins, E) lipoproteins, F) immunoglobulins, G) other plasma proteins, and H) tissue leakage. To clarify the figure, proteins whose abundance made up less than 0.33% of the average total intensity in each condition were pooled into “Other Proteins”. See supplementary proteomics data file for the complete list. N = 3 independent biological replicates for all panels.

**Figure** **6**A illustrates the domains of the proteins the corona on PDMA coated bag and uncoated PVC bags, highlighting key differences in corona composition due to surface chemistry: broadly, uncoated PVC enriched hydrophobic and lipid‐associated proteins, while PDMA coating bound hydrophilic plasma proteins. The primary domains which make up the two coronas differed, with over 40% coagulation proteins on PDMA coating and lipoproteins on uncoated PVC. Proteome dynamics differed between the surfaces as well: PDMA coating saw a peak in acute phase proteins, albumin, and lipoproteins at 4 h, while complement factors and immunoglobulins accumulated between 4–24 h but declined at 7 days. On uncoated PVC, tissue‐associated and coagulation proteins rose sharply within 24 h but decreased by 7 days, alongside increasing complement factors and immunoglobulins (Figure 6A). Despite these differences, similar proportions of immunologically active proteins were observed across different conditions and timepoints.

Figures 6B–H examine the individual proteins in the coronas on PDMA coated bag and uncoated PVC bag and their changes across time. On PDMA coating, over 40% of detected proteins were coagulation‐related, with fibrinogen fragments comprising at least two‐thirds at all time points (Figure 6C). Other fibrinogen‐related proteins were abundant on the surface as well, including coagulation factor XI (FAXI) and kininogen (KNG1). FXI and KNG1 accumulated over time, increasing from 7% and 3% to 10% and 5% between 4 h and 7 days, respectively. While complement proteins made up a similar ratio of total proteins between coated and uncoated units (Figure 6A), PDMA coating accrued lectin pathway‐associated complement factors, namely ficolins 2 and 3, as well as MASP 1 and 2 (Figure 6D). These proteins also accumulated with time, but to a lesser extent than KNG1 and FXI, increasing ≈1% throughout storage. Relatively high amounts of other inflammation‐ and coagulation‐associated proteins, such as HRG and ITIH4 (Figure 6G), were also found on PDMA coating (between 5–10x the % abundance on uncoated PVC bag, respectively).

In contrast, uncoated PVC bag primarily accumulated hydrophobic proteins, with over half being apolipoproteins whose ratios remained stable over time (Figure 6E). There was a sharp rise in cell‐fragment proteins at 24 h, making up 15% of the detected proteins on uncoated PVC bags (Figure 6H). These fragments were primarily actin‐ and dynein‐associated proteins, as well as organelle fragments including ribosomes (Figure 6H). Notably, ≈1% of the proteins detected on uncoated PVC bag were lipopolysaccharide binding protein (LPB, Figure 6B), despite no bacteria being detected in any of the plasma units tested. Uncoated PVC bag surface also accumulated ITIH1 and 2 (Figure 6G), two extracellular matrix‐associated plasma proteins involved in hyaluronan stabilization during inflammation. Taken together, the protein coronas formed on uncoated PVC bag and PDMA coated bag surface differ substantially, with the former accumulating proteins associated with hydrophobic biological roles such as cell membranes and apolipoproteins, whereas the latter accumulated plasma proteins and fibrinogen‐associated factors with changes over storage period.

We further investigated the forces driving the adsorption of some of the most abundant proteins on the PDMA:PDA surface (fibrinogen and FXI) to understand whether their abundance on the protein coronas was due to their affinity for the coating, or for other proteins already adsorbed. In this experiment, fibrinogen (2 mg mL^−1^) and FXI (5 µg mL^−1^) were flowed over QCM chips coated with the PDMA:PDA 15:1 coating, either alone or in combination with plasmas depleted of the respective proteins (Figure S6A, Supporting Information). These experiments revealed that fibrinogen bound to the surface strongly, reaching ≈800 ng cm^−2^ on the surface at equilibrium (Figure S6B, Supporting Information). Interestingly, pre‐treating the surfaces with fibrinogen increased the total quantity of proteins that adsorbed after adding the depleted plasma, resulting in 2000 ng cm^−2^ of total protein, whereas flowing native plasma or pre‐treating the surface with fibrinogen‐depleted plasma before adding fibrinogen both resulted in 1500 ng cm^−2^ (Figure S6C, Supporting Information). Depositing fibrinogen first also increased the resistance of the protein corona to SDS washing when compared to the coronas formed by native plasma, or if depleted plasma was added before fibrinogen. This data suggest that fibrinogen strongly interacts with the coating and enhances the recruitment of other proteins to the surface, as well as the hardness of the corona. FXI also bound with high affinity to the PDMA coating, with a binding capacity of 400 ng cm^−2^ at equilibrium (Figure S6B, Supporting Information). However, its adsorption was reduced >75% when the surface was pre‐treated with FXI‐depleted plasma (100 ng cm^−2^) (Figure S6C, Supporting Information). Pre‐treating the surfaces with FXI did not impact subsequent protein adsorption, suggesting that FXI binds due to its affinity for the coating, but does not drive further protein adsorption.

## 3 Discussion

Platelet activation by synthetic surfaces remains a central issue in the development of blood‐contacting devices and technologies, including platelet storage bags. In this direction, hydrophilic antifouling and antiadhesive coatings hold promise as an efficacious and practical approach to improve the biocompatibility of the surface of these materials in the context of platelet storage. Although some antifouling coatings were shown to reduce platelet activation and improve the platelet storage lesion (PSL) in simplified storage models,^[^ ^32^, ^33^ ^]^ none of the approaches have been deployed in animal studies or clinical testing for eventual clinical use. Our goal was to understand the relationship between PSL and platelet‐surface interactions, and whether manipulation of surface‐platelet interaction using polymer coatings could attenuate the PSL and improve the platelet storage quality when stored for extended periods of time. Thus, we have investigated both the surface protein composition of the bags as well as the quality of the stored platelets at different periods of time under blood banking conditions.

The PSL is thought to be caused by a combination of damage during manufacturing and storage (including agitation and shear forces), the propensity of PVC storage bag surface for protein adsorption and denaturation, oxidative stress and the lack of antioxidants like nitric oxide, and platelet senescence.^[^ ^4^, ^13^, ^60^, ^61^ ^]^ Fibrinogen and other plasma proteins deposited on the PVC surface offer a scaffold onto which platelets can adhere and become activated; stimulated platelets can then activate surrounding cells either by direct interactions through integrins, or by degranulation. Thus, approaches that could decrease platelet adhesion and activation on the bag surface should attenuate both pathways, especially in the acid citrate dextrose (ACD) anticoagulated plasma which inhibits many platelet‐activating factors. However, there is a paucity of research concerning the extent to which these pathways are active in ACD anticoagulated environment of platelet storage bags and multi‐day storage at room temperature (22 ± 2 °C). Although conventional wisdom supports the relationship between protein and platelet‐adhesion‐resistant surfaces and improved platelet storage quality, a nuanced characterization of this pathway's contribution to the PSL is not available. Such information would significantly improve our ability to design novel platelet storage approaches for extending the storage life of platelets and attenuating PSL.

In this research, we demonstrate a library screening and coating methodology in aqueous buffer that works to identify platelet‐biocompatible coatings and translate them on to platelet storage bags. The coating approach is a one‐step dip coating method in aqueous conditions and is amenable to scale‐up. We initially screened a library of ultra‐high molecular weight polymers in various ratios with the PDA binder to identify those which could reduce platelet adhesion and protein adhesion the most (Figure 1). Of the screened polymers, PDMA, PAAm, and PMPC were found to be most effective in ratios of 15:1 with PDA, reducing platelet adhesion >80% after 4 h incubation in buffy coat PCs (Figure 3B) with considerable reduction in the adsorption of fibrinogen and albumin (>99%) compared to uncoated control. The polymers in this group have shown the capacity of reducing platelet adhesion and coagulation activation in the other coating configurations.^[^ ^62^ ^]^ Hydrophilic polymers like PDMA and zwitterionic PMPC are known to improve PVC's hemocompatibility, so their performance here is in line with the findings in the literature.^[^ ^14^, ^21^ ^]^ Platelets adhered to the coated surfaces displayed less shape change and formed smaller aggregates than those on PVC (Figure 1D). When translated into mini‐PVC platelet storage bags, these coatings still significantly reduced platelet adhesion. Furthermore, these thin hydrophilic coatings (≈60–70 nm wet thickness) had no deleterious effects on the cells when screened with a panel of widely accepted assays of platelet quality, and did not compromise gas permeability (Figures 3, 4, 5 Figures 3‐4). Taken together, this approach is appropriate for screening and identifying coatings that are biocompatible with platelets in long‐term storage.

Despite storing the platelets in four bags with distinct surface chemistries (three hydrophilic polymer‐coated and bare PVC), and reducing platelet adhesion significantly, minimal effects were seen in cell quality. One potential explanation for why diminishing platelet adhesion to the bag surfaces had negligible effects is that adhesion did not decrease *enough*. Surface‐adhered platelets release agonists that activate surrounding cells.^[^ ^20^, ^63^ ^]^ These agonists have thresholds of activation, above which platelet activation plateaus.^[^ ^20^, ^63^ ^]^ Thus, if the platelet adhesion in the mini‐bags tested is well above the amount needed to reach this threshold, reducing adhesion would have no effect. While possible, it is surprising that attenuating adhesion by >80% after 4 h and >65% after 7 days (Figures 1C and 3B, respectively) would have no effects on cell storage quality. Conversely, platelet adhesion may be an insignificant parameter: we found ≈4000 platelets adhered per mm^2^ on bare PVC bags, which amounts to less than 0.1% of the total platelets in the storage bags in PVC being adhered to the surface, and less in the coated bags (Figure 3B,C). It is unknown how many platelets must adhere to the surface and degranulate to stimulate the surrounding cells, especially in ACD media. Available data suggests that 5–10% of platelets need to be activated to elicit a procoagulant response in recalcified media,^[^ ^64^, ^65^, ^66^, ^67^ ^]^ so the number of adhered platelets we saw may be insufficient. The data herein proposes that platelet adhesion onto PVC surfaces does not significantly contribute to PSL, despite previous speculation. Instead, these findings point to other effects as primary contributors to the material‐driven sources of the PSL, such as protein adsorption, leaching plasticizers, or inherent changes to the platelets happening due to fact that they are outside their normal physiological environment and in suspension with enough agonists (accumulated during processing at the blood bank) that can initiate irreversible activation processes. Another possibility is that many of the platelets in solution phase might be interacting with the surface via a “*touch and release*” process which activate the platelets similar to coagulation initiation by coagulation factor XII in bulk phase induced by surfaces described recently.^[^ ^62^ ^]^

An important consideration in long‐term platelet storage is the choice of plasticizer: while plasticizer compatibility with platelets is primarily attributed to gas exchange, other factors contribute, such as the material's hydrophobicity, protein fouling, and leaching plasticizers.^[^ ^17^ ^]^ DEHP, the former industry‐standard plasticizer for platelet bags, has deleterious effects on platelets in storage independent of gas exchange.^[^ ^29^, ^30^ ^]^ Previous groups who published on antifouling coatings that improved platelet storage quality either employed the discontinued PVC‐DEHP^[^ ^14^ ^]^ or did not specify.^[^ ^33^ ^]^ Indeed, the success of one previous platelet‐friendly antifouling coating was attributed to the coating's ability to diminish both plasticizer leaching and protein adsorption.^[^ ^14^, ^32^ ^]^ Conversely, we used TOTM‐plasticized PVC because it is one of the most common plasticizers used for PC storage after DEHP's discontinuation due to its relatively low volatility and toxicity,^[^ ^17^, ^68^ ^]^ but no differences were seen. The contribution of plasticizers was not directly investigated in this study, and there is insufficient data in the literature to clearly define the contribution of these different chemicals to the PSL. However, future studies should aim to compare their antifouling surfaces in various platelet bag materials to ensure standardization of results and help delineate which variables are most important to platelet quality.

The current paradigm for generating hemocompatible materials is to minimize protein adsorption, thereby preventing subsequent platelet adhesion and initiation of both coagulation and inflammation cascades. This mechanism is complicated by the presence of ACD in platelet storage units, silencing most stimulatory signals throughout storage. Although groups have generated anti‐adhesive coatings which improve platelet activation levels,^[^ ^14^, ^33^ ^]^ part of this success was attributed to the coating's ability to diminish plasticizer leaching into solution.^[^ ^32^ ^]^ These studies also measured serum protein adsorption, but not plasma proteins, so the extent of their plasma protein adsorption in ACD is unknown. Moreover, recent data indicates that the gold standard of decreasing protein adsorption to reduce platelet and plasma protein activation may not address the root cause of material‐induced coagulation.^[^ ^62^ ^]^ Collectively, the available literature does not clearly demonstrate a relationship between protein adsorption, platelet adhesion and activation, and progression of the PSL in platelets stored in citrated plasma.

The protein corona formed on blood‐contacting surfaces is the primary point of interaction between blood and the material surfaces. Cellular responses to synthetic surfaces in blood are highly dependent on both the quantity and species of proteins adsorbed on the material.^[^ ^56^, ^69^, ^70^, ^71^ ^]^ Thus, two different surfaces which develop similar protein coronas may not differentially affect the platelets they contact.^[^ ^72^, ^73^, ^74^ ^]^ To understand the influence of protein corona on PSL, we performed detailed analysis of proteome on the hydrophilic polymer coated bag versus control uncoated bag under platelet storage conditions (Figures 5 and 6). Proteomic characterization of the protein coronas formed on the PDMA‐coated and uncoated PVC bags returned with stark differences. The adsorption of coagulation and host‐response factors are a common event for blood‐contacting polymers, and known sources of platelet activation.^[^ ^41^ ^]^ The deposition of fibrinogen, coagulation factor XI and kininogen, lectin complement pathway ficolins 2 and 3, and their downstream targets MASP 1 and 2, are therefore unsurprising. Further investigation (Figure S6, Supporting Information) revealed that both FXI and fibrinogen bind to the coatings due in part to their affinity for the coating, and that fibrinogen supports the adsorption of subsequent proteins to the surface. However, the Vroman effect suggests that over time, proteins with high molecular weight (fibrinogen weighing 340 and FXI 160 kDa) and affinity for the coating will accumulate on surfaces, displacing the smaller and more mobile proteins that adsorb first.^[^ ^75^, ^76^ ^]^ Given the citrated plasma environment in the platelet storage bags, it is unlikely that these proteins are being activated by the polymer coating. Thus, it is unclear whether this binding pattern is due to the proteins’ affinity for the coating, or from activation of their biochemical pathways. Conversely, while fibrinogen was abundant (5 to 10% of total protein intensity) on uncoated PVC's surface, more than 50% of the detected proteins were apolipoproteins, and included up to 15% cellular fragments as well. This data agrees with previous investigations into plasma protein adsorption on surfaces, where hydrophobic materials tend to enrich such hydrophobic proteins.^[^ ^77^, ^78^, ^79^ ^]^ The abundance of LBP and C4 fragments, in tandem with the cell fragments and absence of MASP 1 and 2, suggest classical complement pathway activation. In tandem with the significantly reduced platelet adhesion, the data suggests that platelets should be differentially activated by the two surfaces. Our data suggest that the species of proteins adhered, or their quantity, may not be the primary contributor to platelet activation in platelet storage conditions or PSL.

One possibility that unifies these competing mechanisms is the role of protein fold states: platelets are known to show a strong binding preference for denatured or conformation altered proteins, including fibrinogen.^[^ ^74^, ^80^, ^81^ ^]^ Such proteins are also known to activate platelets, although the exact mechanism remains elusive.^[^ ^82^, ^83^, ^84^ ^]^ Denaturation could be occurring on the highly protein‐dense environment of the bag surfaces, providing a consistent source of stimulus to the cells. If platelet contact (as opposed to adhesion) is sufficient for denatured proteins to stress platelets, this may also explain why the various bag materials did not impact platelet storage quality despite significantly reducing adhesion. Denaturation/conformation alteration may also explain why we saw >65% less platelet adhesion on PDMA/PDA, despite detecting triple the amount of fibrinogen on the coated surface when compared to PVC. While this possibility is intriguing, more work in this area is needed to validate it. Future studies should aim to demonstrate both the quantity and fold state of the proteins deposited on the storage bag surfaces, especially throughout the storage period, to hopefully identify which proteomic changes correlate with platelet aberration.

The research herein raises several questions concerning the pathways through which PVC bag surface contributes to the PSL, and the development of next‐generation platelet storage bags. This research also provides new directions to improve cell storage quality. First, platelet adhesion is not well established as an indicator of biocompatibility in platelet storage bags and platelet storage conditions, i.e., room temperature storage. Simply reducing platelet binding may not be an effective approach to improving platelet bags, unless binding can be reduced far below what we achieved here. Although it is understood that protein deposition on PVC surfaces can modulate platelet activity, it is ambiguous whether the species, amount, or fold state of the proteins are the primary contributors to platelet aberrations in citrated plasma media. In this direction, the effects of different bag plasticizers are an important consideration for developing biocompatible surfaces for platelet storage, as well as standardizing the research in this direction. Finally, active strategies to combat the PSL (e.g., introduction of nitric oxide or other antioxidants)^[^ ^13^, ^85^ ^]^ should be explored to directly combat the PSL.

## 4 Experimental Section

### Polymer Synthesis and Coating Development

Poly(*N,N*‐dimethylacrylamide) (PDMA), poly(2‐methacryloyloxyethyl phosphorylcholine), (PMPC) and poly(N‐(tris(hydroxymethyl)methyl) acrylamide (PTHMAM) were synthesized by Atom Transfer Radical Polymerization of their respective monomers and characterized (ref). Polyacrylamide (PAAm, molecular weight kDa) was purchased from Polysciences. Poly(N‐vinylcaprolactam) (PVCL, molecular weight kDa) was purchased from Polymer Source. Polyvinylpyrrolidone (PVP, molecular weight kDa), poly(2‐ethyl‐2‐oxazoline) (PEOx, molecular weight kDa), polyethylene oxide (PEO, molecular weight kDa) dopamine hydrochloride (>97%), sodium periodate (>99%), sodium citrate (>99%) were purchased from Sigma‐Aldrich. LDH Cytotoxicity Colorimetric Assay kit (catalog number K311‐400) was purchased from BioVision.

### Characterization of polymer‐coated surfaces—ATR‐FTIR Attenuated Total Reflectance Fourier Transform Infrared (ATR‐FTIR)

The ATR‐FTIR spectra were collected on a Bruker 670 Tensor‐II (Bruker, Billerica, MA) with a MCT/A liquid nitrogen cooled detector, a KBr beam splitter, and a VariGATR Grazing Angle accessory (Harrick Scientific, Pleasantville, NY). Spectra were recorded at 2 cm^−1^ resolution and 128 scans were collected for each sample. **Scanning Electron Microscopy (SEM)**: A Hitachi SU3500 (Hitachi, Tokyo, JA) SEM (acceleration voltage of 5 kV) was employed. To prepare samples, PVC coupons were adhered onto the aluminum SEM stubs using a conductive carbon double‐side tape. The SEM stub with sample was loaded on a Leica (Wetzlar, GE) sputter coater (working distance: 3 cm and current: 80 mA) for coating the sample with a 20‐nm iridium (Ir) layer.

### Characterization of polymer‐coated surfaces—Atomic Force Microscopy (AFM)

AFM measurements were performed using a commercially available multimode system with a scan range of 130 × 130 µm^2^, controlled by a NanoScope IIIa controller (Digital Instruments, Santa Barbara, CA). Force measurements were performed under PBS buffer in contact mode using a commercially manufactured V‐shaped silicon nitride (Si_3_N_4_) cantilever with gold on the back for laser beam reflection (NP‐S20, Veeco, Plainview, NY). On the tip approach, the onset of the region of constant compliance was used to determine the zero distance, and on retraction, the region in which force was unchanged was used to determine the zero force. The rate of the tip‐sample approach or retraction was typically 1 µm s^−1^. The raw AFM force data were converted into force versus separation using custom Matlab v.5.3 software. The software converts the cantilever deflection versus linear voltage displacement transformer signal into restoring force versus tip substrate separation using user input trigger and spring constant values. The previously published protocols were followed for the calculation of equilibrium thickness, adhesive force and rupture distance.^[^ ^44^ ^]^

### Preparation of Hydrophilic Polymer/PDA Coating Library on PVC Platelet Bag Coupons

Polymer solutions were prepared at a concentration of 36 mg mL^−1^ in citrate buffer (50 mm, pH 5). Dopamine and sodium periodate solutions were freshly prepared at a concentration of 12 and 28 mg mL^−1^, respectively before each experiment. The coating solution was prepared by firstly mixing PDMA and dopamine solution at a volume ratio of 5:1. The sodium periodate was added immediately into the mixed solution with a volume ratio of 0.2:1 to the dopamine solution. The PVC sheet (TOTM‐plasticized PVC, Tianhe Pharmaceutical Co., China) was cut into a coupon size with diameter 13 mm and left into the wells of 24‐well plate. The coating solution (720 µL) was introduced into each well. The plate was left on an orbital shaker at 50 rpm for 2 h. The coated coupon was thoroughly washed with Mill‐Q water and dried with argon. Coupons and the wells were subsequently sterilized by washing with 70% ethanol and rinsed 3 times with sterile PBS (10 mm).

### Short‐term anti‐adhesive Coating screening—Blood Collection

Blood was collected from consented healthy donors at the Centre for Blood Research, the University of British Columbia, into 3.8% sodium‐citrated vacutainers. Platelet was obtained by centrifugation of citrate whole blood at 800 rpm (123 *g*) for 15 min. Blood collection and the protocols used in the current studies were approved by the Clinical Research Ethics Board of the University of British Columbia (H20‐00084) and written consent from donors was obtained accordingly.

### Short‐term anti‐adhesive Coating screening—Incubation

PVC coupons coated with hydrophilic polymer/PDA were transferred to the wells of 24‐well plate. Platelet rich plasma (1 mL) was added to each well. The plate was left on an obituary shaker with gentle shaking at 50 rpm for 4 h at room temperature (22 °C). After incubation, the coupons were gently rinsed with sterile PBS buffer (10 mm) three times. **For SEM**: Coupons from each polymer/PDA coating group were immersed into 1 mL 2.5% glutaraldehyde in PBS for 1 h. After fixation, coupons were washed with PBS buffer twice, then with distilled water twice. The coupons were left at room temperature to dry overnight. **For quantification of adhered platelets**: Five coupons from each polymer/PDA coating group were immersed into 500 µL 1.8% (V/V) Trixon‐100 solution for 1 h at 37 °C. The quantification of LDH released from lysed platelet adhered onto the surface was evaluated by LDH cytotoxicity assay (Abcam, Cambridge, UK). A standard curve of LDH generated from a known number of platelets was employed to quantify the number of platelets based on LDH activity in the unknown samples.

### Blood Product Processing, Sample Preparation, and Experimental Setups—Development of Coating on mini‐platelet Storage Bags

For this study, mini‐platelet storage bags (45 mL, Figure S2, Supporting Information) were custom made from PVC plasticized with TOTM by Tianhe Pharmaceutical Co., China (Figure S1, Supporting Information). Three mini‐bags were coated for each polymer composition (PDMA, PAAM, and PMPC), and 3 were left uncoated as controls. To coat the mini‐bags, solutions of the polymer (36 mg mL^−1^), dopamine (12 mg mL^−1^), and sodium periodate (28 mgmL^−1^) in 50 mm sodium citrate (pH 5) were prepared fresh for each new set of bags. To prepare the coating solution, polymer and dopamine solutions were mixed in a ratio of 15:1. Sodium periodate solution was added in a ratio of 1:0.2 to dopamine, mixed by vortex, sonicated 15 s to remove bubbles, and immediately added to the mini‐bags, taking care to remove all air bubbles via syringe and bag manipulation. The bags were set on orbital shakers (15 oscillations per min) for 2 h, and flipped after 1 h. After incubation, the reaction solution was removed, and the bags were washed 5x with 10 mm PBS (45 mL each wash). The bags were then sterilized by adding 70% ethanol and shaken for 5 min. In a biological safety cabinet, the ethanol was removed, and bags were washed 5 x with sterile PBS (45 mL per wash).

### Blood Product Processing, Sample Preparation, and Experimental Setups—Platelet Concentrate Preparation

This study was approved by the Research Ethics Boards of CBS (2021.046) and the University of British Columbia (H21‐02554). Informed consent was obtained from all healthy volunteers prior to donation. Whole blood donations were collected by CBS. The manufacture of buffy coat PCs in plasma (blood group matched) was performed by the NetCAD development laboratory of CBS (Vancouver, BC, Canada) using standard operating procedures 22 h after collection. After a 6 h rest period, 2 platelet units were pooled and split into two identical platelet units using a TSCD‐II tube welder (Terumo, Tokyo, JA).^[^ ^52^, ^53^ ^]^ Coated and uncoated control mini‐bags were filled with 45 mL PC, and stored under constant agitation (Helmer Scientific, PC1200‐Pro Platelet Incubator, Noblesville, IN). Platelet units were sampled through a coupling port (Fresenius Kabi, Bad Homburg, Germany) using a sterile syringe in a biological safety cabinet. The units were sampled on days 1, 2, 4, and 7 for storage for testing. **For detailed explanation of the statistical approach,** see the “Statistical Analysis” section at the end of the Materials and Methods section. **Autologous plasma**: To generate autologous platelet‐poor plasma for clotting experiments, after the 6 h post‐manufacturing rest period on the day of collection, PC were centrifuged at 2000 g for 15 min at 22 °C, and aliquots were stored at ‐70 °C. Individual aliquots were thawed at 37 °C for sure throughout the storage study.

### In Vitro Quality Measurements of Stored Platelets—Automated Quality Assessments

Platelet count was determined using a hematology analyzer (Sysmex XN‐550, Sysmex Corporation, Kobe, Japan). The blood gases and metabolites (pCO_2_, pO_2_, glucose, lactate, and pH) were quantified via blood gas analyzer (GEM Premier 4000, Instrumentation Laboratory, Bedford, MA). The sterility of all platelet units was tested on day 7 by plating aliquots 500 µL on blood agar plates (VWR, Mississauga, ON, Canada), followed by a 24 h incubation at 37 °C.

### In Vitro Quality Measurements of Stored Platelets—Flow Cytometry

Platelet activation was quantified by measuring the expression of P‐selectin (CD62P). Here, the platelet sample was diluted to 200 × 10^9^ L^−1^ in PBS buffer (10 mm). A 45 µL sample was then incubated for 30 min with phycoerythrin‐labeled anti‐CD62P and FITC‐labelled anti‐CD41 as a platelet marker (Beckman‐Coulter, Mississauga, ON, Canada). In parallel, platelets were also treated with 10 µM ADP before antibody incubation to measure the degranulation response of the platelets. Indications of cell death were tracked by measuring the exposure of phosphatidylserine, using FITC‐labelled annexin V. Platelet samples were diluted to 100 × 10^9^ L^−1^ in HEPES Ca^2+^ buffer and incubated with annexin‐V‐FITC (BD Biosciences) for 30 min. All samples were then analyzed on a flow cytometer (FACSCanto II, BD Biosciences, Mississauga, ON, Canada). The sterility of all platelet units was tested on day 7 by plating aliquots on blood agar plates (VWR, Mississauga, ON, Canada), followed by a 24 h incubation at 37 °C.

### In Vitro Quality Measurements of stored Platelets—Rotational Thromboelastometry

The ability of the platelets to support coagulation was evaluated using Rotational Thromboelastometry (ROTEM) (Werfen, Barcelona, Spain). Platelets were diluted in autologous plasma to 300 and 100 × 10^9^ L^−1^ for EXTEM and INTEM tests, respectively. Three hundred microliters of autologous plasma were added to the ROTEM cups, and 20 µL each of START‐TEM and either EXTEM or INTEM were added. Reactions were run for 1 h.

### In Vitro Quality Measurements of Stored Platelets—Morphology Scoring

Platelets were visualized using phase‐contrast light microscopy to measure changes to cellular morphology using the Kunicki method.^[^ ^54^ ^]^ Briefly, platelets were sampled and fixed with 2% paraformaldehyde (PFA) for at least 30 min and imaged using phase contrast microscopy at 90x magnification. Structural changes in platelets during storage were evaluated using Kunicki's morphology score. Ten images were captured per sample, and images were randomly selected for morphology scoring until 200 platelets were scored for each. Kunicki score was calculated according to the following formula: 4 x (% Discs) + 2 x (% Spheres) + 1 x (% Dendrites) + 0 x (% Balloons).

### In Vitro Quality Measurements of Stored Platelets—Post‐Storage Bag Handling

After collecting all data from day 7 of storage, the bags were processed to collect SEM images and quantify the adhered platelets. All subsequent steps were performed in a biological safety cabinet with sterile reagents and equipment: After storage, the PCs were removed from the bags, which were then gently washed 3 times with 45 mL PBS (10 mm, pH 7.4). The bags were then cut open, and 8 square coupons with length of 1 cm were cut from the bags. **For SEM**: Platelet coupons were fixed with 2.5% glutaraldehyde and washed with PBS and water, as previously described. **For LDH assay**: Coupons were incubated with 400 µL 1.8% triton 100x in PBS for 1 h at 37 °C to lyse the adhered cells. After lysis, the LDH activity was quantified by LDH cytotoxicity assay (Abcam, Cambridge, UK), and the number of adhered platelets was determined using a standard curve, as described previously.

### Proteomic Analysis of Protein Corona in Platelet Storage Bags—Experimental Set‐Up

To characterize the protein corona that forms in the storage units, and how the anti‐adhesive coatings affect the corona, a proteomic analysis was performed. Autologous plasma was generated from pooled‐and‐split PCs and depleted of PMVs as outlined in “Plasma Preparation” above. Forty‐five mL of plasma was kept in either uncoated units or coated with the 15:1 PDMA/PDA coating formulation. Proteomic characterization was performed at timepoints of 4, 24 h, and 7 days. At each timepoint, plasma extracted from the bags was also streaked on agar plates to ensure units were not contaminated.

### Proteomic Analysis of Protein Corona in Platelet Storage Bags—Tryptic Digest & Protein Preparation

At each timepoint, in a biological safety cabinet, the plasma was removed, and the bags were washed with 10 mm PBS as previously described to remove residual plasma (Figure S2, Supporting Information). In a dust‐free fume hood, the bags were cut open, and 2 cm^2^ circles were drawn on the interior using a hydrophobic marker (ImmEdge H‐4000, Vector Laboratories, Newark, NY), within which the tryptic digest was performed. These circles were filled with digestion buffer (150 uL ammonium bicarbonate containing 1 ug trypsin). After 90 min, the reaction was stopped by adding 5% formic acid. The digestion drop was removed and prepared for LC/MS using iST kits for biological fluids (PreOmics, Munich, Germany). Briefly, after reaction stoppage, the solutions in the cartridges were removed by centrifugation at 3800 *g*, and peptides were washed with accompanying “Wash” buffers, before being eluted using “Elution” buffer. Peptides were dried by vacuum evaporator and stored in 50 µL “LC‐LOAD” buffer.

### Proteomic Analysis of Protein Corona in Platelet Storage Bags—LC‐MS Analysis

For a detailed description of the LC‐MS protocol used, see Supplementary Materials. Briefly, samples were reconstituted in 0.5% acetonitrile, 0.1% formic acid. One hundred ng of peptides were separated using an Easy‐nLC 1200 with a 25 cm Aurora Series C18 column at 40 °C. Analysis was performed over 60 min using a 0.25 µL min^−1^ flow rate, with a gradient from 2% to 95% buffer B:buffer A, where A was 0.1% formic acid and 2% acetonitrile in water, while B was 0.1% formic acid and 80% acetonitrile in water. Peptides were analyzed on a Orbitrap Exploris 480 (Thermo Fisher, Waltham, MA) in data‐independent acquisition mode (DIA) using full MS scans at 60 000 resolution and DIA fragment scans at 15 000 resolution. Ion source voltage was 1900 V with 290 °C ion transfer tube temperature. A 28% normalized collision energy was used for fragmentation. Scan ranges were m/z 380 to 985 for MS, and m/z 145 to 1450 for DIA. Data were collected with Thermo Scientific Xcalibur (version 4.7). Data were analyzed library‐free on DIA‐NN (version 1.8.1; PMID:31 768 060) using a Homo sapiens database from Uniprot (reviewed sequences only, downloaded from Uniprot) and common contaminants (211 entries).^[^ ^86^ ^]^ Search parameters included trypsin/P specificity, one missed cleavage, and settings for N‐terminal M excision and cysteine carbamidomethylation, with a 1% precursor FDR. Quantification was performed with high precision, cross‐run normalization, and smart profiling mode for library generation. The LC‐MS analysis was further detailed in the Supplementary Materials.

### Proteomic Analysis of Protein Corona in Platelet Storage Bags—Proteomic Data Processing

Metadata about the identified proteins was retrieved from UniprotKB by searching for the peptides identified by mass spectrometry in Uniprot's “ID Mapping” function. Isoelectric points of the peptides were calculated from ExPASy.^[^ ^87^ ^]^ Hits from other species and common contaminants (e.g., keratin) were removed before analysis. For the classification of proteins in Figure 6, plasma and tissue‐associated proteins were first separated by “subcellular compartment” labels from the metadata. Proteins were separated into functional families using Gene Ontology's “Biological Processes” tags (coagulation, acute phase, etc.). Because apolipoproteins and antibodies wwere abundant in the plasma and carry out many different roles, these proteins were pooled by family directly rather than sorting them by biological process. For the statistical approach, see the “Statistical Analysis” section below.

### Plasma Preparation, Handling, and Quantification—Plasma Preparation

For all studies on plasma performed in this research, plasma was prepared as follows: Plasma was retrieved by spinning pooled‐and‐split PCs at 2000 g for 15 min at 22 °C. To minimize the confounding effects of platelet microvesicles (PMVs) on the proteomic data and adsorbed protein quantification, PMVs were centrifuged out by spinning plasma at 15 000 g for 30 min (Figure S2A, Supporting Information). PMVs were quantified as described in Chen et al. (2018).^[^ ^50^ ^]^

### Plasma Preparation, Handling, and Quantification—Platelet Bag Coating and Handling

To prepare the mini‐bags for plasma storage and quantification, 45 mL PVC‐TOTM mini‐bags were either left uncoated or coated with 15:1 PDMA:PDA coating, as previously described. Three set of bags were coated for each timepoint tested in this study (4, 24 h, and 7d). Autologous plasma from the pooled‐and‐split platelet units was also prepared and spun to remove microvesicles as previously described. Coated and uncoated mini‐bags were filled with 45 mL plasma and put into storage on standard platelet shakers at 22 °C.

### Plasma Preparation, Handling, and Quantification—Protein Extraction

To extract the proteins adsorbed on uncoated PVC and PDMA coated bags after 4, 24 h, and 7d of storage, coated and uncoated bags were treated as follows: In a biological safety cabinet, plasma from the bags was removed via syringe. Residual plasma was then washed out by filling the bags with 45 mL of 10 mm P BS pH 7.4 and placing on a shaker for 5 min. This process was repeated 3 times. To extract adsorbed proteins from the bag surfaces, an SDS buffer was used: 10% SDS (BIO‐RAD, Hercules, CA), with 1 mm ethylenediaminetetraacetic acid (EDTA) and phenylmethylsulfonyl fluoride (PMSF) protease inhibitors (both from Sigma‐Aldrich, St. Louis, MO), pH 7.4. Bags were filled with 45 mL of SDS buffer and placed in 50 °C baths for 30 min. Every 5 min, bags were manually agitated for 30 s to ensure thorough mixing. After 30 min, the SDS solution was removed from the bags, and the amount of protein within was quantified. Quantification of released proteins: All equipment and reagents in this section were purchased from Bio‐Rad. Protein quantification was performed by gel densitometry. A standard curve of plasma with known amounts of protein was run on each gel to calculate the concentration of protein in the eluted samples. Samples were mixed with SDS sample buffer (62.5 mm Tris pH 6.8, 10% glycerol, 3% SDS, 0.0167% bromophenol blue) boiled (95 °C for 5 min), separated by SDS‐PAGE on TGX Stain‐Free FastCast gels (10% acrylamide), and transferred to nitrocellulose membranes (Transblot Turbo Transfer System). Membranes were imaged using a ChemiDoc Imager, and densitometry was performed with ImageLab software.

### Quartz Crystal Microbalance—Experimental Setup

To evaluate the adsorption behavior of fibrinogen (Fg) and coagulation factor XI (FXI) onto the PDMA‐PDA surface, quartz crystal microbalance (QCM) was used to track the adsorption of these proteins in real time. QCM chips were coated with PDMA:PDA at a 15:1 ratio as described earlier, and loaded into the QCM (Biolin Scientific, SWE). The chips were equilibrated by flowing buffer until a stable baseline was reached, defined by reaching zero slope in the resonance frequency signal. Protein adsorption was monitored by tracking the shifts in resonance frequencies across the coated surface. Recombinant FXI (Cedarlane, Burlington, ON, CA) was prepared in citrate phosphate dextrose buffer (CPD, pH 7.4) to inhibit the enzyme's activity, while purified Fg (2 mg mL^−1^) and human serum albumin (HSA, 2 mg mL^−1^) were prepared in PBS (pH 7.4). Native platelet poor plasma (received fresh from CBS), Fg‐depleted and FXI‐depleted plasmas (Affinity Biologicals, Ancaster, ON, CA), were frozen at ‐70 °C, and aliquots were thawed for each experiment.

### Quartz Crystal Microbalance—Experimental Workflow

A standardized workflow was followed for each experiment. For all experiments, the flow rate was 50 µL min^−1^. For each step of the QCM workflow, the buffers and proteins were run until the signal stabilized (resonance frequency slopes = 0) (Figure S6A, Supporting Information). The experimental workflow was as follows: (1) Baseline stabilization with buffer until QCM frequency slope = 0. (2) Flow the first protein of interest over the chip (Protein #1 Added step). (3) Buffer wash to remove loosely associated proteins and quantify the firmly adsorbed proteins (Buffer Wash #1 step). (4) If applicable, flow of the second protein, followed by another buffer wash (Protein Added #2 and Buffer Wash #2 steps). (5) After completing the necessary protein and buffer steps, all samples were washed with 2% SDS to wash away proteins bound by hydrophobic interactions (SDS Wash step). (6) After washing with SDS, a final buffer wash was performed to remove the residual SDS adsorbed onto the surface (Buffer Wash #3 step). See Figure S6A (Supporting Information) for visualization of the stepwise QCM process.

### Statistical Analysis—Platelet Experiments

Statistical analysis was performed using GraphPad Prism 10 (GraphPad Software, Inc., La Jolla, CA) for all chemistry and platelet‐related work. For each assay, the means of the three technical replicates for each biological replicate were computed. The means and standard deviations of the biological replicates were then used for statistical analysis. Figures show means, and error bars show standard deviations. Unless otherwise specified, all platelet and chemistry‐related statistical analyses were carried out using two‐way analysis of variance (ANOVA) with repeated measures when comparing all coatings in each experiment. Timepoints were used as the within‐subject factors, and coatings were used to compare between groups. In these experiments, multiple comparisons were made between coating conditions at each timepoint. Multiple comparisons were corrected using Tukey's correction. Because the sample size for the platelet experiments (Figure 3, 4, 5) was small (n = 3), formal tests of the ANOVA assumptions were interpreted cautiously. In addition to these tests, the normality and variance of the data were evaluated by visual inspection of the QQ and residual plots. Violations of sphericity were corrected using the Greenhouse‐Geisser correction.

### Statistical Analysis—Proteomic Analysis

Unless otherwise specified, all proteomic work was performed using R Version 4.3.3. Percent abundance of the proteins was calculated using the LFQ intensities relative to the total sum of protein LFQ intensities for each group in each biological replicate. Average relative intensities for the three biological replicates are shown for each group. Proteins were considered for the analysis if they were detected in at least 2 of 3 biological replicates. To generate the completed dataset, missing data were imputed by Bayesian principal component analysis with 5 principal components, using the package pcaMethods.^[^ ^88^ ^]^ The data was log_2_ transformed, and base R was used to perform statistical tests: The correlation tests were performed using Spearman's correlation test, and comparisons on the amount of proteins were performed using 2‐way ANOVA with Tukey's correction for multiple comparisons. Data manipulation was performed with base R as well as dplyr and tidyr packages.^[^ ^89^, ^90^ ^]^ Panels in Figure 6 Figure 5 were generated using ggplot2.^[^ ^91^ ^]^ Preparation of the data for the bar graphs in Figure 6 was performed in R with the packages listed above, and the final figure was assembled in GraphPad Prism.

## Conflict of Interest

The authors declare no conflict of interest.

## Supporting information



Supporting Information

Supporting Information

## Data Availability

Please confirm the selected template for your Data Availability Statement below, which will be published as part of your article if it is accepted for publication. The data that support the findings of this study are openly available in ProteomeXChange at https://doi.org/10.25345/C5445HQ71, reference number 96889.
