# Supplementary material for: Investigation on the Influence of Protein Corona and Platelet Adhesion on Storage Bag Surface on the Platelet Storage Lesion
Source: Adv Healthc Mater. 2025 Jun 23;14(23):2501217. doi: 10.1002/adhm.202501217 (PMC12417748; doi:10.1002/adhm.202501217)
Supplement: Supplementary file 1 — Supporting Information [file ADHM-14-0-s001.docx]

**Investigation on the Influence of Protein Corona and Platelet Adhesion on Storage Bag Surface on the Platelet Storage Lesion**

Nicolas Pereyra^a,b^, Kai Yu^a,c^, Jason Rogalski^d^, Taylor Da Silva^c^, Parisa Golesorkhi^c^, Madeleine Goldstein^e^, Dana V. Devine^a,b,c^, Jayachandran N. Kizhakkedathu^a,c,f,*^

a. Centre for Blood Research and Life Sciences Institute, University of British Columbia, 2350 Health Sciences Mall, Vancouver, BC, V6T 1Z3. b. Department of Biochemistry & Molecular Biology, University of British Columbia, 2350 Health Sciences Mall, Vancouver, BC, V6T 1Z3. c. Department of Pathology and Laboratory Medicine, University of British Columbia, 2350 Health Sciences Mall, Vancouver, BC, V6T 1Z3, d. Centre for High Throughput Biology, Michael Smith Laboratories, University of British Columbia, 2125 East Mall, University of British Columbia, Vancouver, BC, Canada V6T 2A1, e. Department of Cellular and Physiological Sciences, University of British Columbia, 2350 Health Sciences Mall, University of British Columbia, Vancouver, BC, Canada V6T 1Z3. f. School of Biomedical Engineering, University of British Columbia, Vancouver, BC, Canada V6T 1Z3.

* Correspondence

Email: [jay@pathology.ubc.ca](mailto:jay@pathology.ubc.ca)

**Supplementary Information**


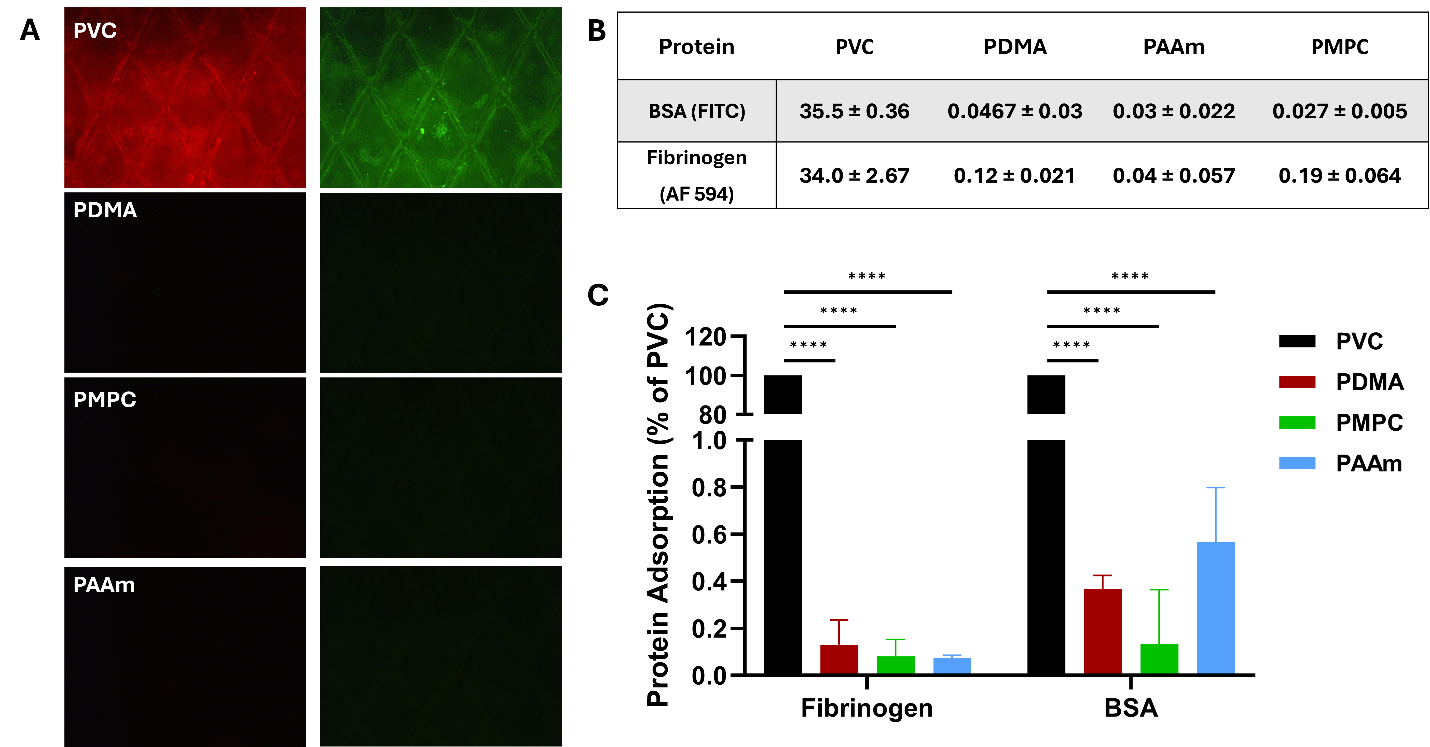


**Figure S1. Anti-protein fouling performance of the top coating formulations for long-term platelet storage.** Coupons cut from PVC minibags (**Fig. S2**) were coated with either PDMA, PAAm, or PMPC in 15:1 ratio with dopamine as described in the Methods section. Afterwards, the coupons were incubated with either 0.5 mg/mL AF-594-labelled fibrinogen, or 1 mg/mL FITC-labelled BSA, and incubated for 30 min in 24-well plates at 22 ^o^C. **(A)** Fluorescent intensity of either AF 594-labelled fibrinogen or FITC-labelled BSA found on the surface of coupons after 30 min incubation. **(B)** Fluorescent intensity of the proteins adhered to the coupon surfaces, measured by densitometry. Figure shows mean ± SD. **(C)** Percentage of protein found on the surface of the different coupons when compared to PVC (100%). Statistical comparisons were performed using unpaired t-tests. (****) indicates p>0.0001. N=3 independent experiments.

**
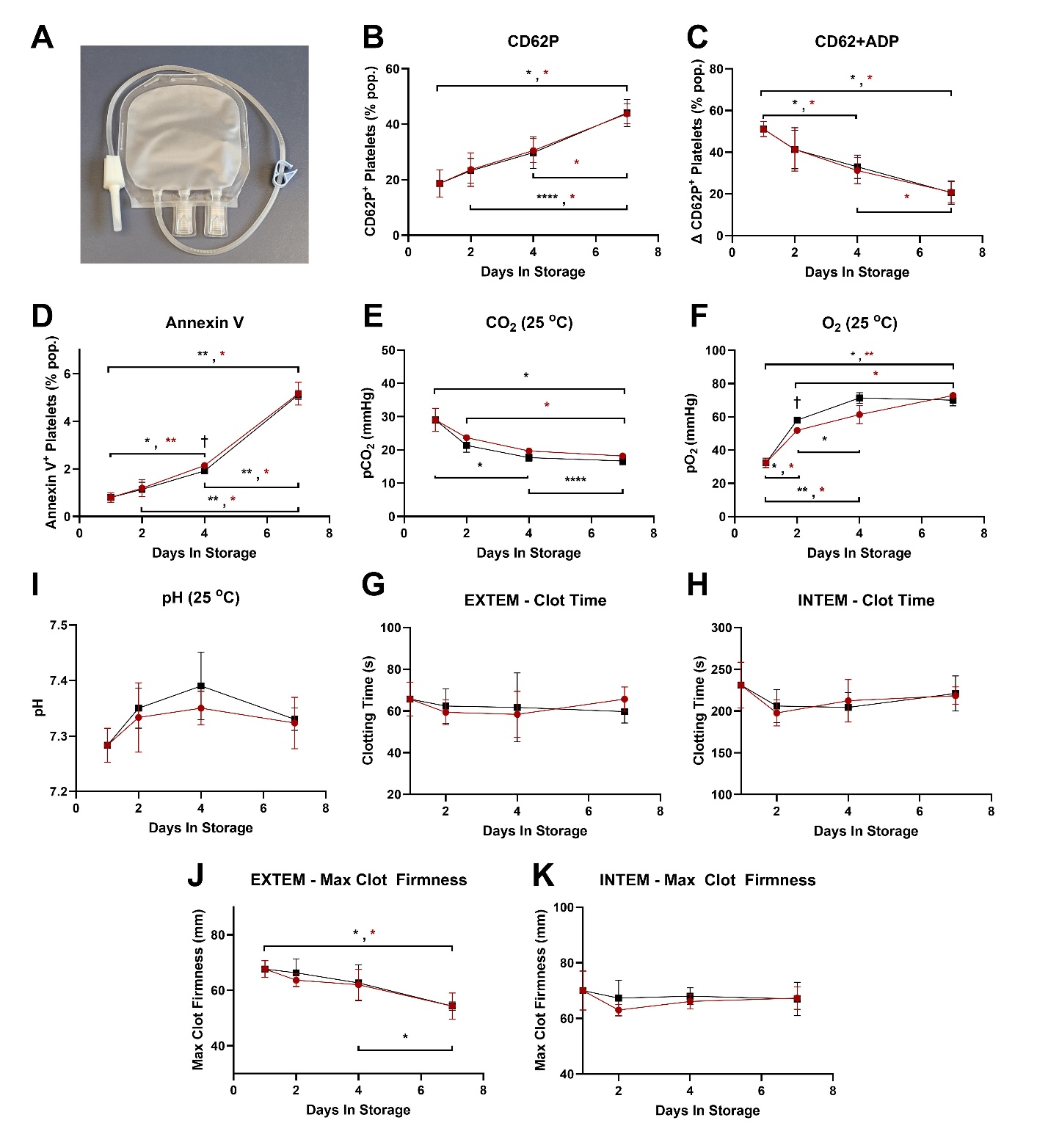
**

**Figure S2. Non-inferiority testing of PVC-TOTM minibags compared to standard platelet storage bags. (A)** Proprietary minibags made of PVC plasticized with Tris (2-ethylhexyl) Trimellitate (TOTM) were manufactured by Tianhe Pharmaceutical Co. for this study. Platelet concentrates received from CBS were pooled-and-split and were placed into either full 350 mL storage units used at CBS, or in minibags, and placed into CBS-standard storage for 7 days. On days 1, 2, 4 and 7, platelets were assayed for quality using industry-standard protocols. **(B-D)** Platelet activation was evaluated using flow-cytometric quantification of CD62P and Annexin V. Platelet responsiveness was measured by subtracting CD62P display at rest from CD62P after treatment with 10 mM ADP. **(E-G)** Platelet metabolic health was assessed by blood gas metrics pH, O_2_, and CO_2_. (**H-K)** Platelet clotting response via extrinsic and intrinsic coagulation activation was evaluated using ROTEM. Statistical significance across time is denoted by the system: For comparisons within a given group across time, significance is denoted by asterisk (*) and the color corresponds with the group compared (minibags in red, CBS-standard bags in black). (†) denotes differences between CBS bags and minibags within a given timepoint. (*) <0.05, (**) <0.01, (***) <0.001, and (†) > 0.05. N=3 independent biological replicates.


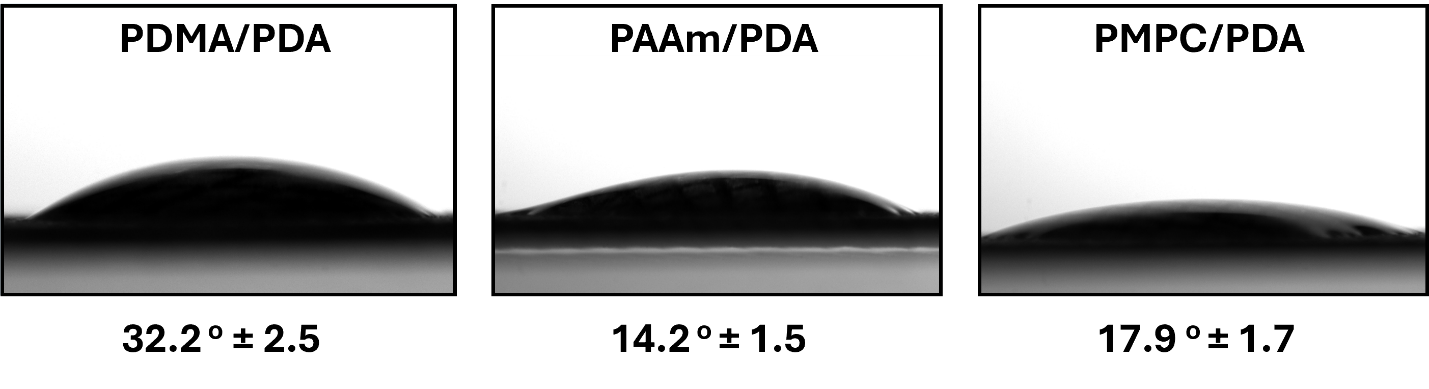


**Figure S3. Water contact angle on coated PVC.** Water contact angle was measured on coupons cut from minibags coated with either PDMA, PAAm, or PMPC/PDA. Water contact angle is listed as angle ± SD. N=3 independent experiments.


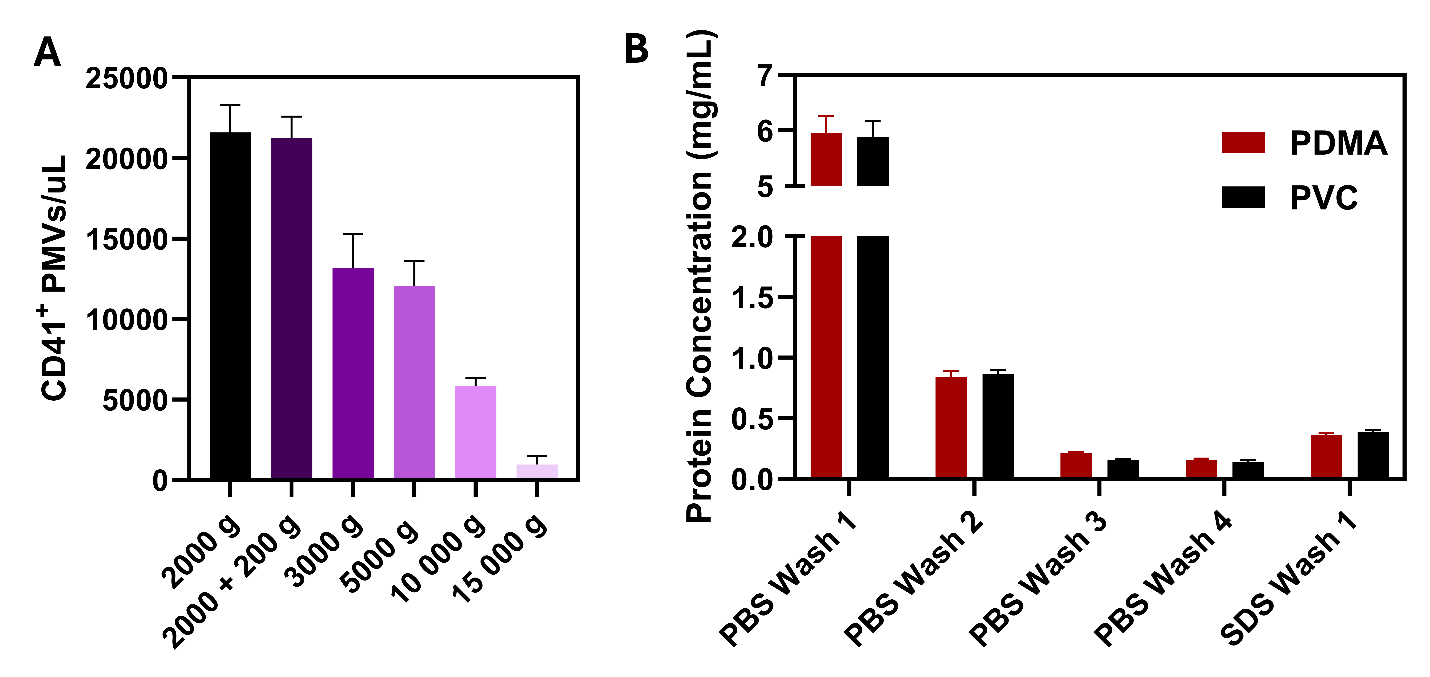


**Figure S4. Optimization of plasma processing steps. (A)** Number of PMVs found in plasma after a series of spins. Plasma derived from platelet concentrates was spun at one of the following g forces for 30 min: 2000, 2000 + 200 (two separate spins), 3000, 5000 g, 10 000, or 15 000. **(B)** Amount of protein extracted from each wash step using PBS and SDS buffers. Plasma was stored in 45 mL minibags that were either uncoated (PVC) or coated with PDMA 15:1 PDA for 24 h. N=3 independent biological replicates for each.

**Figure S5. Quantification of adsorbed proteins on PVC and PDMA surfaces after 7-day storage.** The amount of adsorbed proteins on the surfaces of the bare and PDMA-coated minibags after 7-day storage was determined by gel densitometry. Proteins were eluted using 45 mL of 10% SDS buffer with EDTA and PMSF protease inhibitors and incubating for 30 min at 50 ^o^C (see Materials for detailed protocol). **No statistically significant differences were seen.** N=3 independent biological replicates.

**
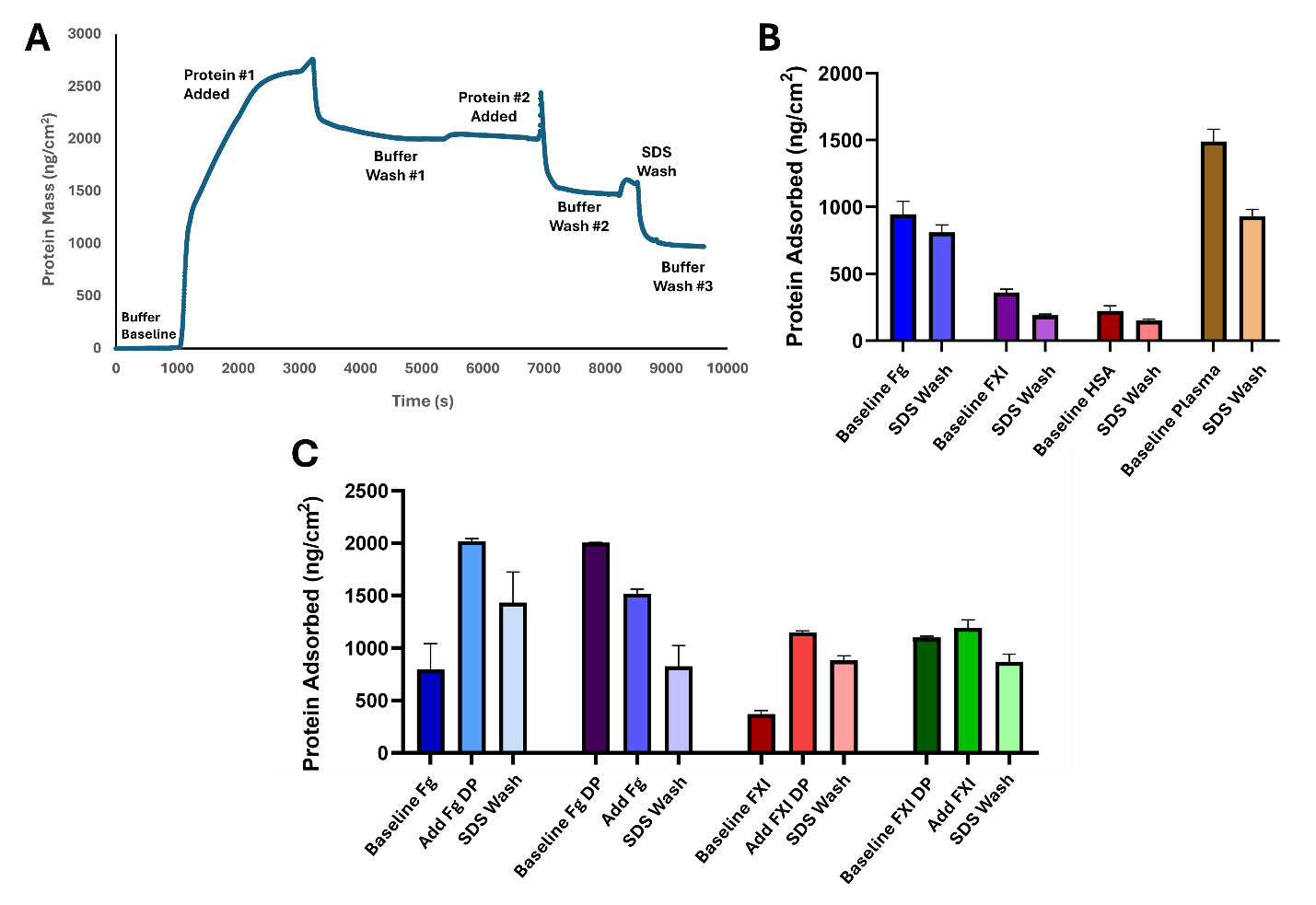
**

**Fig. S6. Quantity of protein adsorbed on PDMA-PDA coated chips. (A)** Workflow for the QCM experiments. Each step in the workflow was performed until the QCM frequency curves (proportional to mass of protein adsorbed) reached equilibrium (approximately 20 minutes for each step). **(B)** Quantity of Fibrinogen (Fg), Factor XI (FXI), Human Serum Albumin (HSA), and Plasma (native, undiluted) adsorbed to the PDMA-PDA-coated QCM chips at equilibrium after Buffer Wash #1, as well as after SDS Wash. **(C)** Sequential adsorption experiments involving FXI and Fg with FXI- and Fg-depleted plasmas (DP). For each pair of bars, the left bar (“Baseline”) represents the first protein added after buffer wash, while the right bar (“Add”) represents the second protein adsorbed after buffer wash. The washing steps were performed to ensure quantification of stably bound protein. Protein adsorption was calculated by averaging the QCM signal across the plateau following each addition. N=2 independent experiments per condition.

**Table S1. Molecular weight and polydispersity of polymers tested.** Molecular weights for polymers are listed as either average molecular weight (Mn), weight-average molecular weight (Mw), or viscosity-average molecular weight (Mv), as appropriate. PAAm, PVP, and PEO were purchased from vendors, and polydispersity was labelled as ‘not defined’ (ND).

| **Polymer** | **Molecular Weight (Daltons)** | **Polydispersity** |
| --- | --- | --- |
| **PDMA** | 795,000 (Mn) | 1.2 |
| **PMPC** | 1,310,000 (Mw) | 1.22 |
| **PAAm** | 400,000 - 1,000,000 (Mw) | ND |
| **PDEA** | 900,000 (Mn) | 1.2 |
| **PVP** | 1300,000 (Mw) | ND |
| **PVCL** | 354,000 (Mn) | 4.6 |
| **PTHMAM** | 632,000 (Mn) | 1.66 |
| **PEOX** | 500,000 (Mw) | 3.5 |
| **PEO** | 900,000 (Mv) | ND |

**Table S2. Statistically significant comparisons and corresponding P values for platelet storage data.** Statistical comparisons herein correspond to Figures 3 and 4 of the main manuscript body. All comparisons shown are within a given coating across time. The timepoints compared are noted in the ‘Comparisons’ column, where the number next to ‘D’ denotes days of storage being compared. For all tests, N=3 independent biological replicates, and degrees of freedom = 2. P values were adjusted using Tukey Statistical significance is denoted by star system in ‘Summary’ column, where: (*) p<0.05, (**) p<0.01, (***) p<0.001, (****) p<0.0001. See the ‘Statistical Approach’ section of the Materials and Methods for details on the statistical approach.

| **Variable** | **Coating** | **Comparisons** | **Mean 1** | **Mean 2** | **SE of diff.** | **q** | **Summary** | **Adjusted P Value** |
| --- | --- | --- | --- | --- | --- | --- | --- | --- |
| **CD62P + ADP** |  |  |  |  |  |  |  |  |
|  | **PDMA** | D1 vs. D2 | 54.7 | 38.8 | 2.2 | 10.3 | * | 0.0452 |
|  |  | D1 vs. D4 | 54.7 | 27.3 | 0.9 | 41.7 | ** | 0.0019 |
|  |  | D1 vs. D7 | 54.7 | 16.8 | 2.3 | 23.2 | ** | 0.0095 |
|  |  | D2 vs. D4 | 38.8 | 27.3 | 1.4 | 11.9 | * | 0.0342 |
|  |  | D2 vs. D7 | 38.8 | 16.8 | 1 | 31.8 | ** | 0.0049 |
|  |  | D4 vs. D7 | 27.3 | 16.8 | 1.4 | 10.7 | * | 0.0421 |
|  | **PAAM** | D1 vs. D2 | 54.7 | 41.2 | 0.6 | 31.6 | ** | 0.005 |
|  |  | D1 vs. D4 | 54.7 | 32.2 | 1.5 | 20.8 | * | 0.0116 |
|  |  | D1 vs. D7 | 54.7 | 17 | 1.7 | 31.7 | ** | 0.0049 |
|  |  | D2 vs. D7 | 41.2 | 17 | 1.5 | 23.1 | ** | 0.0096 |
|  | **PMPC** | D1 vs. D2 | 54.7 | 40.3 | 1.1 | 18 | * | 0.0154 |
|  |  | D1 vs. D4 | 54.7 | 28.3 | 0.8 | 49.3 | *** | 0.0007 |
|  |  | D1 vs. D7 | 54.7 | 18.3 | 2 | 25.7 | ** | 0.0078 |
|  |  | D2 vs. D4 | 40.3 | 28.3 | 1.1 | 15 | * | 0.022 |
|  |  | D2 vs. D7 | 40.3 | 18.3 | 1.8 | 17.4 | * | 0.0163 |
|  |  | D4 vs. D7 | 28.3 | 18.3 | 1.3 | 11.2 | * | 0.0386 |
|  | **PVC** | D1 vs. D2 | 54.7 | 40.5 | 0.2 | 87 | **** | <0.0001 |
|  |  | D1 vs. D4 | 54.7 | 31 | 1.2 | 28.1 | ** | 0.0065 |
|  |  | D1 vs. D7 | 54.7 | 15.7 | 2.5 | 21.8 | * | 0.0107 |
|  |  | D2 vs. D4 | 40.5 | 31 | 1.1 | 12.5 | * | 0.0311 |
|  |  | D2 vs. D7 | 40.5 | 15.7 | 2.6 | 13.4 | * | 0.0272 |
| **Annexin V** |  |  |  |  |  |  |  |  |
|  | **PDMA** | D1 vs. D7 | 0.8 | 4.8 | 0.5 | 11.8 | * | 0.0347 |
|  |  | D2 vs. D7 | 1.1 | 4.8 | 0.4 | 12.4 | * | 0.0316 |
|  | **PAAM** | D1 vs. D7 | 0.8 | 4.9 | 0.3 | 23.2 | ** | 0.0095 |
|  |  | D2 vs. D7 | 1.1 | 4.9 | 0.2 | 29.5 | ** | 0.0059 |
|  | **PMPC** | D1 vs. D7 | 0.8 | 5.1 | 0.5 | 13.1 | * | 0.0285 |
|  |  | D2 vs. D7 | 1.1 | 5.1 | 0.4 | 13.6 | * | 0.0264 |
|  | **PVC** | D1 vs. D7 | 0.8 | 5.2 | 0.5 | 13.7 | * | 0.0263 |
|  |  | D2 vs. D7 | 1.1 | 5.2 | 0.5 | 12.6 | * | 0.0308 |
| **pO2** |  |  |  |  |  |  |  |  |
|  | **PDMA** | D1 vs. D4 | 39 | 60 | 2.5 | 11.8 | * | 0.0349 |
|  |  | D1 vs. D7 | 39 | 62.6 | 3.1 | 10.8 | * | 0.0415 |
|  | **PAAM** | D1 vs. D7 | 39 | 69.3 | 3.1 | 13.9 | * | 0.0256 |
|  | **PMPC** | D1 vs. D7 | 39 | 71.8 | 1.7 | 26.9 | ** | 0.0071 |
|  | **PVC** | D1 vs. D7 | 39 | 64.9 | 2.4 | 15.2 | * | 0.0213 |
| **pH** |  |  |  |  |  |  |  |  |
|  | **PDMA** | D1 vs. D2 | 7.2 | 7.4 | 0 | 35.6 | ** | 0.0035 |
|  |  | D1 vs. D4 | 7.2 | 7.4 | 0 | 14.9 | * | 0.0221 |
|  |  | D4 vs. D7 | 7.4 | 7.3 | 0 | 30 | ** | 0.0056 |
|  | **PAAM** | D1 vs. D2 | 7.2 | 7.4 | 0 | 25.7 | ** | 0.0078 |
|  |  | D1 vs. D4 | 7.2 | 7.4 | 0 | 13.6 | * | 0.0266 |
|  | **PMPC** | D1 vs. D2 | 7.2 | 7.4 | 0 | 18.3 | * | 0.0149 |
|  |  | D1 vs. D4 | 7.2 | 7.4 | 0 | 15.7 | * | 0.0201 |
|  | **PVC** | D1 vs. D2 | 7.2 | 7.4 | 0 | 14.8 | * | 0.0224 |
|  |  | D1 vs. D4 | 7.2 | 7.4 | 0 | 13.8 | * | 0.0258 |
|  |  | D4 vs. D7 | 7.4 | 7.3 | 0 | 19.8 | * | 0.0128 |
| **CO2** |  |  |  |  |  |  |  |  |
|  | **PDMA** | D1 vs. D2 | 44.3 | 22.2 | 1.1 | 28.6 | ** | 0.0063 |
|  |  | D1 vs. D4 | 44.3 | 17.1 | 0.6 | 65.5 | **** | <0.0001 |
|  |  | D1 vs. D7 | 44.3 | 16 | 0.7 | 57.8 | *** | 0.0002 |
|  |  | D2 vs. D4 | 22.2 | 17.1 | 0.6 | 11.7 | * | 0.0356 |
|  |  | D2 vs. D7 | 22.2 | 16 | 0.6 | 15 | * | 0.022 |
|  |  | D4 vs. D7 | 17.1 | 16 | 0.1 | 14.1 | * | 0.0246 |
|  | **PAAM** | D1 vs. D2 | 44.3 | 22 | 0.6 | 54.7 | *** | 0.0003 |
|  |  | D1 vs. D4 | 44.3 | 16.8 | 0.6 | 70.1 | **** | <0.0001 |
|  |  | D1 vs. D7 | 44.3 | 15.4 | 0.6 | 73.5 | **** | <0.0001 |
|  |  | D2 vs. D4 | 22 | 16.8 | 0.3 | 25.1 | ** | 0.0082 |
|  |  | D2 vs. D7 | 22 | 15.4 | 0.3 | 31.5 | ** | 0.005 |
|  |  | D4 vs. D7 | 16.8 | 15.4 | 0.1 | 14 | **** | <0.0001 |
|  | **PMPC** | D1 vs. D2 | 44.3 | 21.8 | 0.6 | 54.3 | *** | 0.0004 |
|  |  | D1 vs. D4 | 44.3 | 17.1 | 0.6 | 65.5 | **** | <0.0001 |
|  |  | D1 vs. D7 | 44.3 | 14.7 | 0.5 | 82.4 | **** | <0.0001 |
|  |  | D2 vs. D4 | 21.8 | 17.1 | 0.4 | 17.2 | * | 0.0169 |
|  |  | D2 vs. D7 | 21.8 | 14.7 | 0.3 | 34.2 | ** | 0.004 |
|  | **PVC** | D1 vs. D2 | 44.3 | 20.9 | 0.9 | 36.5 | ** | 0.0032 |
|  |  | D1 vs. D4 | 44.3 | 16.7 | 0.5 | 76.8 | **** | <0.0001 |
|  |  | D1 vs. D7 | 44.3 | 15.1 | 0.3 | 140.6 | **** | <0.0001 |
| **Glucose** |  |  |  |  |  |  |  |  |
|  | **PDMA** | D1 vs. D4 | 310 | 269.7 | 4.8 | 12 | * | 0.034 |
|  |  | D1 vs. D7 | 310 | 233 | 9.3 | 11.7 | * | 0.0358 |
|  |  | D2 vs. D4 | 290.9 | 269.7 | 2.8 | 10.8 | * | 0.0418 |
|  | **PAAM** | D1 vs. D7 | 310 | 231.8 | 6.8 | 16.4 | * | 0.0185 |
|  |  | D2 vs. D4 | 296.4 | 272.1 | 2.3 | 14.7 | * | 0.0228 |
|  |  | D2 vs. D7 | 296.4 | 231.8 | 3 | 30.6 | ** | 0.0054 |
|  |  | D4 vs. D7 | 272.1 | 231.8 | 1.6 | 36.2 | ** | 0.0033 |
|  | **PMPC** | D1 vs. D7 | 310 | 231.9 | 10.3 | 10.8 | * | 0.0416 |
|  |  | D2 vs. D7 | 296.1 | 231.9 | 8.9 | 10.2 | * | 0.0466 |
|  |  | D4 vs. D7 | 272.3 | 231.9 | 3.7 | 15.4 | * | 0.0209 |
|  | **PVC** | D1 vs. D7 | 310 | 236.7 | 4.4 | 23.8 | ** | 0.009 |
|  |  | D2 vs. D7 | 301.4 | 236.7 | 7.5 | 12.2 | * | 0.0327 |
| **Lactate** |  |  |  |  |  |  |  |  |
|  | **PDMA** | D1 vs. D7 | 9.2 | 15.4 | 0.8 | 11.6 | * | 0.036 |
|  |  | D2 vs. D7 | 9.3 | 15.4 | 0.8 | 11.5 | * | 0.0367 |
|  |  | D4 vs. D7 | 11.1 | 15.4 | 0.4 | 14.4 | * | 0.0237 |
|  | **PAAM** | D1 vs. D7 | 9.2 | 15.3 | 0.7 | 12.4 | * | 0.0316 |
|  |  | D2 vs. D4 | 9.5 | 11.3 | 0.2 | 10.2 | * | 0.0466 |
|  |  | D2 vs. D7 | 9.5 | 15.3 | 0.6 | 12.6 | * | 0.0306 |
|  |  | D4 vs. D7 | 11.3 | 15.3 | 0.4 | 14 | * | 0.025 |
|  | **PMPC** | D1 vs. D7 | 9.2 | 15.7 | 0.7 | 12.9 | * | 0.0296 |
|  |  | D2 vs. D4 | 9.4 | 11.2 | 0.2 | 10.6 | * | 0.0431 |
|  |  | D2 vs. D7 | 9.4 | 15.7 | 0.7 | 13.1 | * | 0.0284 |
|  |  | D4 vs. D7 | 11.2 | 15.7 | 0.5 | 13.8 | * | 0.0258 |
|  | **PVC** | D1 vs. D7 | 9.2 | 16.1 | 0.7 | 14 | * | 0.025 |
|  |  | D2 vs. D7 | 9.6 | 16.1 | 0.7 | 13.7 | * | 0.026 |
|  |  | D4 vs. D7 | 11.4 | 16.1 | 0.4 | 18.7 | * | 0.0144 |
| **CD62P** |  |  |  |  |  |  |  |  |
|  | **PDMA** | D1 vs. D4 | 20.4 | 37.9 | 2.3 | 11 | * | 0.0402 |
|  |  | D1 vs. D7 | 20.4 | 53.9 | 2.5 | 18.7 | * | 0.0143 |
|  |  | D2 vs. D4 | 26.5 | 37.9 | 0.3 | 50.3 | *** | 0.0006 |
|  |  | D2 vs. D7 | 26.5 | 53.9 | 3.2 | 12.1 | * | 0.0331 |
|  | **PAAM** | D1 vs. D7 | 20.4 | 52.3 | 1.3 | 36.1 | ** | 0.0033 |
|  |  | D2 vs. D7 | 25 | 52.3 | 1.4 | 26.8 | ** | 0.0072 |
|  | **PMPC** | D1 vs. D7 | 20.4 | 53.6 | 0.7 | 65.6 | **** | <0.0001 |
|  |  | D2 vs. D4 | 26.4 | 38.5 | 1.7 | 10 | * | 0.0481 |
|  |  | D2 vs. D7 | 26.4 | 53.6 | 1.6 | 24.7 | ** | 0.0084 |
|  | **PVC** | D1 vs. D4 | 20.4 | 39 | 2.6 | 10.1 | * | 0.0468 |
|  |  | D1 vs. D7 | 20.4 | 53.2 | 1.7 | 28 | ** | 0.0066 |
|  |  | D2 vs. D4 | 24.8 | 39 | 1.9 | 10.5 | * | 0.0438 |
|  |  | D2 vs. D7 | 24.8 | 53.2 | 1.1 | 37.2 | ** | 0.003 |
| **Morphology Score** | |  |  |  |  |  |  |  |
|  | **PDMA** | D1 vs. D4 | 296.7 | 261.8 | 4.9 | 10 | * | 0.0484 |
|  |  | D1 vs. D7 | 296.7 | 210.3 | 5.8 | 21.1 | * | 0.0114 |
|  |  | D2 vs. D7 | 296.2 | 210.3 | 0.9 | 139.2 | **** | <0.0001 |
|  |  | D4 vs. D7 | 261.8 | 210.3 | 6.9 | 10.5 | * | 0.0438 |
|  | **PAAM** | D1 vs. D4 | 296.7 | 259.9 | 2.5 | 20.8 | * | 0.0117 |
|  |  | D1 vs. D7 | 296.7 | 207.9 | 3.6 | 35.4 | ** | 0.0036 |
|  |  | D2 vs. D4 | 290.2 | 259.9 | 3.3 | 13 | * | 0.0291 |
|  |  | D2 vs. D7 | 290.2 | 207.9 | 0.8 | 147.8 | **** | <0.0001 |
|  |  | D4 vs. D7 | 259.9 | 207.9 | 2.7 | 27.6 | ** | 0.0068 |
|  | **PMPC** | D1 vs. D4 | 296.7 | 256.8 | 3.7 | 15.2 | * | 0.0213 |
|  |  | D1 vs. D7 | 296.7 | 212.2 | 5.2 | 23.1 | ** | 0.0096 |
|  |  | D2 vs. D4 | 291.9 | 256.8 | 3.7 | 13.6 | * | 0.0267 |
|  |  | D2 vs. D7 | 291.9 | 212.2 | 4.8 | 23.5 | ** | 0.0092 |
|  |  | D4 vs. D7 | 256.8 | 212.2 | 2.1 | 30.7 | ** | 0.0053 |
|  | **PVC** | D1 vs. D7 | 296.7 | 200.9 | 5.6 | 24.1 | ** | 0.0089 |
|  |  | D2 vs. D7 | 291 | 200.9 | 4.3 | 29.7 | ** | 0.0058 |
| **E-MCF** |  |  |  |  |  |  |  |  |
|  | **PDMA** | D1 vs. D4 | 64.67 | 54.33 | 0.6939 | 21.06 | * | 0.0114 |
|  |  | D1 vs. D7 | 64.67 | 48.89 | 1.16 | 19.23 | * | 0.0135 |
|  |  | D2 vs. D7 | 60.11 | 48.89 | 0.4444 | 35.71 | ** | 0.0035 |
|  | **PAAM** | D1 vs. D4 | 64.67 | 55.56 | 1.252 | 10.29 | * | 0.0455 |
|  |  | D1 vs. D7 | 64.67 | 49.83 | 0.8553 | 24.53 | ** | 0.0085 |
|  |  | D2 vs. D7 | 61.33 | 49.83 | 1.11 | 14.66 | * | 0.0229 |
|  |  | D4 vs. D7 | 55.56 | 49.83 | 0.4747 | 17.05 | * | 0.0171 |
|  | **PMPC** | D1 vs. D4 | 64.67 | 53.78 | 1.544 | 9.976 | * | 0.0483 |
|  |  | D1 vs. D7 | 64.67 | 48.33 | 1.54 | 15 | * | 0.0219 |
|  |  | D2 vs. D4 | 59.78 | 53.78 | 0.3333 | 25.46 | ** | 0.008 |
|  |  | D2 vs. D7 | 59.78 | 48.33 | 0.294 | 55.06 | *** | 0.0003 |
|  |  | D4 vs. D7 | 53.78 | 48.33 | 0.1111 | 69.3 | **** | <0.0001 |
|  | **Bare PVC** | D1 vs. D4 | 64.67 | 55.67 | 1.072 | 11.88 | * | 0.0345 |
|  |  | D1 vs. D7 | 64.67 | 51.33 | 1.018 | 18.52 | * | 0.0146 |
|  |  | D4 vs. D7 | 55.67 | 51.33 | 0.5092 | 12.04 | * | 0.0336 |
| **Platelet Count** |  |  |  |  |  |  |  |  |
|  | **PDMA** | D1 vs D7 | 850.7 | 790.6 | 60.11 | 5.807 | * | 0.0230 |
|  | **PMPC** | D1 vs D7 | 844.4 | 798.8 | 45.67 | 0.1925 | **** | <0.0001 |
|  | **Bare PVC** | D2 vs D4 | 832 | 803.7 | 28.33 | 7.506 | * | 0.0191 |

**General LC/MS Methodology**

The following protocol was used to prepare samples and perform LC/MS, as well as subsequent data analysis. This is a copy of the shared document standard for The University of British Columbia’s Mass Spectrometry core. Experiments were performed exactly as described.

# **Sample preparation**

Samples were reconstituted in 0.5% acetonitrile, 0.1% formic acid. Sample concentration was measured using NanoDrop One (Thermo Scientific) with the A205 Scope method (absorbance at 205nm, baseline correction at 340nm).

# **Instrument Analysis**

## **Exploris 480 Mass Spectrometer (**ThermoFisher, Waltham, MA, USA)

### **LC method: 60min gradient (standard LC method used for most sample types)**

100ng of peptides were injected and separated on-line using Easy-nLC 1200 (Thermo Fisher Scientific) with Aurora Series analytical column, (25cm x 75μm 1.6μm C18; Ion Opticks, Parkville, Victoria, Australia). The analytical column was heated to 40°C using an integrated column oven (PRSO-V2, Sonation, Biberach, Germany). Buffer A consisted of 0.1% aqueous formic acid and 2% acetonitrile in water, and buffer B consisted of 0.1% aqueous formic acid and 80% acetonitrile in water. A standard 60min gradient was run from 2% B to 20% B over 46 min, then to 32% B over 15 min, then to 50% B from 61 to 66min, then to 95% B over 5 min, held at 95% B for 8 min, then dropped to 3% B over 2 min, held at 3% B for 6 min. The analysis was performed at 0.25 μL/min flow rate. The Easy-nLC thermostat temperature was set at 7°C.

### **MS Method**

**Exploris 60min HeLA 250nl/min DIA** (default DIA method used on Exploris)

The peptides were analyzed with an Orbitrap Exploris 480 mass spectrometer (Orbitrap Exploris^TM^ 480, Thermo Fisher Scientific). The Nanospray Flex^TM^ ion source was operated at 1900 V spray voltage and ion transfer tube was heated to 290°C. During analysis, the Orbitrap Exploris 480 was operated in a data-independent (DIA) mode. The MS and MS/MS spectra were collected in positive mode. Full scans MS resolution was set to 60,000 with 300% normalized automatic gain control (AGC) target, 50% RF lens, 25 ms maximum injection time and scan range from m/z 380 Th to m/z 985 Th. DIA fragment spectra were collected at a resolution of 15,000, normalized AGC target of 2000%, maximum injection time of 40 ms, scan range from m/z 145 Th – m/z 1450 Th. Isolation windows of m/z 10 Th were used with an overlap of m/z 1 Th. Normalized collision energy was set to 28%.

### **Software information**

Data was collected with Thermo Scientific Xcalibur (version 4.7).

# **Data Analysis**

### **Library-free Data-Independent Acquisition by Neural Networks (DIA-NN)**

All acquired data was searched library-free on DIA-NN (version 1.8.1; PMID:31768060) against a database consisting of the Homo sapiens fasta (reviewed sequences only, downloaded from Uniprot) and common contaminants (211 entries). Fasta digest and deep learning-based spectra, RTs and IMs prediction were enabled using trypsin/P protease specificity and 1 missed cleavage. Other search parameters include N-terminal M excision and Cysteine carbamidomethylation. Peptide length ranged 7-30, precursor charge ranged 2-4, precursor m/z ranged 300-1200, and fragment ion m/z ranged 200-1800. Precursor FDR was set to 1%, with 0 for settings ‘mass accuracy’, ‘MS1 accuracy’ and ‘scan window’. Settings ‘heuristic protein inference’, ‘use isotopologues’, ‘match between run (MBR)’, and ‘no shared spectra’ were all enabled. ‘Gene’ was chosen for protein inference parameter along with ‘double-pass mode’ for neural network classifier. Robust LC (high precision) was used for quantification strategy, RT-dependent mode for cross-run normalization, and smart profiling mode for library generation.
